# Making target sites in large structured RNAs accessible to RNA-cleaving DNAzymes through hybridization with synthetic DNA oligonucleotides

**DOI:** 10.1093/nar/gkae778

**Published:** 2024-09-09

**Authors:** Connor Nurmi, Jimmy Gu, Amal Mathai, John D Brennan, Yingfu Li

**Affiliations:** Department of Biochemistry and Biomedical Sciences, McMaster University, Ontario L8S 4L8, Canada; Biointerfaces Institute, McMaster University, Ontario L8S 4L8, Canada; Department of Biochemistry and Biomedical Sciences, McMaster University, Ontario L8S 4L8, Canada; Department of Biochemistry and Biomedical Sciences, McMaster University, Ontario L8S 4L8, Canada; Biointerfaces Institute, McMaster University, Ontario L8S 4L8, Canada; Department of Biochemistry and Biomedical Sciences, McMaster University, Ontario L8S 4L8, Canada; Biointerfaces Institute, McMaster University, Ontario L8S 4L8, Canada; Department of Biochemistry and Biomedical Sciences, McMaster University, Ontario L8S 4L8, Canada

## Abstract

The 10–23 DNAzyme is one of the most active DNA-based enzymes, and in theory, can be designed to target any purine-pyrimidine junction within an RNA sequence for cleavage. However, purine-pyrimidine junctions within a large, structured RNA (lsRNA) molecule of biological origin are not always accessible to 10–23, negating its general utility as an RNA-cutting molecular scissor. Herein, we report a generalizable strategy that allows 10–23 to access any purine-pyrimidine junction within an lsRNA. Using three large SARS-CoV-2 mRNA sequences of 566, 584 and 831 nucleotides in length as model systems, we show that the use of antisense DNA oligonucleotides (ASOs) that target the upstream and downstream regions flanking the cleavage site can restore the activity (*k*_obs_) of previously poorly active 10–23 DNAzyme systems by up to 2000-fold. We corroborated these findings mechanistically using in-line probing to demonstrate that ASOs reduced 10–23 DNAzyme target site structure within the lsRNA substrates. This approach represents a simple, efficient, cost-effective, and generalizable way to improve the accessibility of 10–23 to a chosen target site within an lsRNA molecule, especially where direct access to the genomic RNA target is necessary.

## Graphical Abstract

**Graphical Abstract:**
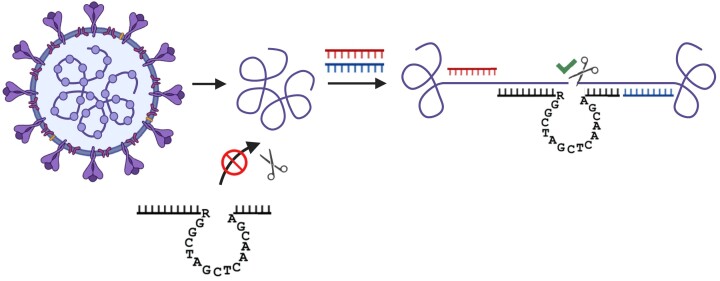

## Introduction

RNA-cleaving enzymes comprised entirely of DNA (DNAzymes) have been used in a myriad of therapeutic and diagnostic applications (1–7). The 10–23 DNAzyme is the most efficient RNA-cleaving DNAzyme (RCD) reported, which contains a catalytic core of 15 nucleotides flanked by substrate recognition arms that selectively bind an RNA sequence through Watson-Crick base pairing (8,9). The 10–23 DNAzyme cleaves most effectively at purine-pyrimidine junctions, particularly for AU and GU sites (10). While 10–23 can achieve high catalytic performance with short RNA transcripts (11,12), reduced activity is often reported for larger, more structured RNA (lsRNA) substrates such as messenger RNAs (13,14), presumably due to extensive secondary and tertiary structures that make access to the target site difficult. For example, we analyzed 230 different 10–23 DNAzymes targeting lsRNA transcripts from the SARS-CoV-2 RNA genome in a previous study and found that nearly 90% possessed little to no RNA cleavage activity (<20% cleaved after 10 min) (15). The issue of target site inaccessibility is not exclusive to the 10–23 DNAzyme, as it also prevalent for other widely studied DNAzymes such as 8–17 (8,16), and also impacts various other classes of functional nucleic acids including ribozymes (17,18) and RNA aptamers (19), as well as CRISPR-associated endonuclease (Cas) enzymes (20,21).

Several strategies have improved the accessibility of 10–23 to structured target sites of lsRNA, which include increasing reaction temperature, pH, magnesium concentration and substrate recognition arm length, or using 10–23 with modified nucleic acids (22–29). However, manipulation of reaction conditions is generally not amenable to therapeutic applications, while longer binding arms could slow the rate of release of the cleavage product for use in rapid diagnostic tests. Substitution of modified nucleic acids has been shown to improve RNA hybridization efficiency for lsRNA and stability in biological samples (23,24,26,30–34), but these modified sequences need lengthy trial-and-error studies for optimization, expensive nucleic acid synthesis, and can be difficult to generate for different lsRNA targets. An alternative approach is to assess the accessibility of potential target sites using RNA structure prediction software, though accuracy decreases with increasing RNA sequence length (35). Experimental approaches have also been used to map accessible sites within lsRNAs; however, such methods are costly, time consuming and impractical for the rapid development of *in-vitro* diagnostics or therapeutics (14,36–38).

One solution to improve DNAzyme accessibility to lsRNA transcripts is with DNA antisense oligonucleotides (ASOs) that hybridize to upstream and/or downstream regions near the cleavage site to alter local secondary or tertiary structures around this site. ASOs were first used for this purpose to improve target site accessibility of the hammerhead ribozyme (39) and since then, have been used to alter lsRNA structure in several contexts. However, many studies possess drawbacks that limit ASOs as a general-purpose strategy to improve DNAzyme accessibility to lsRNA for practical applications.

First, many studies used lsRNA with an experimentally elucidated structure, such as 16S ribosomal RNA (40–46) and transfer-messenger RNA (47,48), which enabled more effective ASO design. In certain cases, such as during the outbreak of SARS-CoV-2, rapid *in-vitro* diagnostics for viral genomic RNA must be developed without experimentally derived genomic structures, which can take years to become available (49). Second, no mechanistic data has been reported that shows a clear reduction in lsRNA target site structure upon ASO addition, which would serve to corroborate the fluorescence output of an lsRNA-targeting DNA probe, or kinetic observations of a lsRNA-cleaving enzyme. Third, ASOs must function across a variety of target sites in order to be used as a general-purpose strategy to improve accessibility to lsRNA, however many studies tested only one target site within a single lsRNA (39,50–54). Finally, no studies showed that ASOs improved the accessibility of *trans*-acting DNAzymes to lsRNA substrates, only *trans*-acting ribozymes (39,50,51) and DNA or RNA probes (40–48,52–57), which interact differently with the lsRNA target. In particular, ribozymes form a more stable RNA-RNA duplex compared to DNA-RNA interactions (58) and can better access structured lsRNA target sites, while for non-catalytic DNA probes, only partial hybridization to the lsRNA is often satisfactory to generate a fluorescent signal above background (41). Conversely, for *trans*-acting DNAzymes, complete hybridization to the lsRNA substrate is absolutely necessary for full activity, with shorter hybridization regions resulting in a significant loss in activity (14,27). Given these drawbacks, a general-purpose strategy to improve the accessibility of trans-acting DNAzymes to lsRNA is necessary, and we hypothesize that using ASOs is the most effective and amenable strategy for practical applications.

Herein, we examined the effects of ASOs on five 10–23 DNAzymes (two with high activity, three with low activity) that targeted five different AU sites located within three SARS-CoV-2 lsRNA transcripts, which were chosen from the 230 DNAzyme set that was previously tested against different cleavage sites of several large SARS-CoV-2 mRNA transcripts (15). ASOs were used in two distinct strategies, the first employing rational design where ASOs bind the upstream and downstream sequences of a chosen target site within a specific lsRNA, and the second involving experimental screening, with a series of ASOs that bind to more distal regions from the target site to identify the most efficient ASOs for improving 10–23 accessibility. With the rational design approach, we observed a restoration in lsRNA cleavage activity for each of the three low activity 10–23 DNAzyme systems tested. Use of an ASO identified through the experimental screening approach further improved the cleavage activity of one DNAzyme relative to rationally designed ASOs. In-line probing of ASO-lsRNA pairs showed a clear reduction in RNA secondary structure at the 10–23 DNAzyme binding site upon ASO addition, confirming the role of ASOs in improving DNAzyme cleavage activity. Overall, these results show the potential of using ASOs as a simple, general-use strategy for improving DNAzyme-based diagnostic assays or therapeutics that utilize an lsRNA as the target.

## Materials and methods

### SARS-CoV-2 RNA preparation and γ-^32^P ATP radiolabeling

The three lsRNAs used in this study were prepared from relevant DNA templates, which were amplified by polymerase chain reaction (PCR) and purified using the New England Biolabs Monarch PCR and DNA clean-up kit. The three SARS-CoV-2 lsRNA substrates were lsRNA-1 (the 584-nt transcript made from the *NSP8* gene covering nucleotides 12 098–12 679 of the wildtype SARS-CoV-2 genome), lsRNA-2 (the 831-nt transcript from the *ORF3a* gene covering nucleotides 25 393–26 220 of the wildtype SARS-CoV-2 genome) and lsRNA-3 (the 566-nt transcript from the *Spike* gene covering nucleotides 24 108–24 665 of the wildtype SARS-CoV-2 genome). Purity of the PCR product was verified by agarose gel electrophoresis and the concentration was determined by *A*_260_ measurement using a NanoVue Plus spectrophotometer. Each PCR-produced SARS-CoV-2 DNA product was transcribed to the corresponding lsRNA in the following reaction conducted at 37°C for 2 h: 100 units of T7 RNA Polymerase, 2 pmol DNA template, 5 mM DTT, 1 × T7 RNA transcription buffer (20 mM Tris–HCl pH 7.9, 3 mM MgCl_2_, 5 mM DTT, 5 mM NaCl and 1 mM spermidine), 0.2 units of pyrophosphatase, 2 mM nucleotide triphosphate (NTP) mixture and 40 units of RiboLock (all from Thermo Scientific). Afterwards, 5 units of DNase I was added and incubated for an additional 20 min at 37°C. Each transcribed lsRNA was purified by denaturing polyacrylamide gel electrophoresis (PAGE) with 8 M urea, imaged by UV shadow and excised. The gel fragment was crushed, soaked in a 1 × elution buffer (200 mM NaCl, 10 mM Tris–HCl pH 7.5 and 1 mM EDTA pH 8.0) and shaken at room temperature for 20 min. EtOH precipitation was then performed to concentrate the lsRNA. The lsRNA pellet was resuspended in nuclease-free H_2_O, and spectrophotometrically quantified. For the relevant 25-nt RNA substrates, sequences were ordered directly from Integrated DNA Technologies and used as received.

All RNA substrates were 5′-radiolabeled with ^32^P as follows: First, 2 pmol RNA was added to 1 unit of alkaline phosphatase (FastAP, ThermoFisher Scientific) in 10 μl of 1 × FastAP buffer (10 mM Tris–HCl pH 8.0, 5 mM MgCl_2_, 500 mM KCl, 0.02% Triton X-100 and 0.1 mg/ml BSA; ThermoFisher Scientific) and incubated for 30 min at 37°C. After the addition of 5 μl of 3 M NaOAc, the dephosphorylated RNA was treated by addition of an equal volume of phenol-chloroform-isoamyl alcohol 25:24:1 pH 6.7/8.0 followed by vortexing. The mixture was centrifuged for 2 min, and the top aqueous layer was removed. The RNA was then washed twice with 50% chloroform and the resulting aqueous layer was concentrated by EtOH precipitation. The dried RNA pellet was resuspended in a solution containing ∼10 μCi γ-^32^P ATP and 10 units of polynucleotide kinase (PNK, ThermoFisher Scientific) in 10 μl of 1 × PNK buffer A (50 mM imidazole-HCl pH 6.4, 18 mM MgCl_2_, 5 mM DTT, 0.1 mM spermidine and 0.1 mM ADP; ThermoFisher Scientific) and incubated for 20 min at 37°C. The phosphorylated RNA was then purified by denaturing PAGE containing 8 M urea and the gel was exposed on an Amersham Biosciences storage phosphor screen. The screen was imaged on an Amersham Typhoon 9200 scanner with a photo-multiplier tube sensitivity of 4000 V. The 5′-radiolabeled ^32^P RNA was excised, crushed, soaked in 1 × elution buffer and EtOH precipitated.

### 10–23 DNAzyme kinetics reactions

All 10–23 DNAzyme kinetics experiments were conducted at room temperature (23°C) in duplicate using a single-turnover, discontinuous enzymatic assay format where individual timepoints were taken from a reaction mixture containing 500 pM 10–23 DNAzyme and ∼1 pM 5′-^32^P labeled RNA substrate in 10 μl of 1 × buffer 1 (50 mM 4-(2-hydroxyethyl)-1-piperazineethanesulfonic acid (HEPES) pH 7.4, 10 mM MgCl_2_ and 100 mM NaCl) and mixed with 10 μl of 2 × quenching buffer (10% sucrose, 0.5 × TBE, 30 mM EDTA and 0.14 mg/ml bromophenol blue and xylene cyanol) to stop the reaction on ice. Before initiation of the reaction by addition of 1 × buffer 1, it was heated for 1 min at 90°C and cooled for 10 min to facilitate uniform folding of the 10–23 DNAzyme, enabling more efficient hybridize to the RNA substrate. After taking a t = 0-min timepoint, 1 × buffer 1 was added and individual timepoints were taken at *t* = 0.5, 1, 2, 5, 10, 15, 30, 45, 60 and 90 min for reactions with the 25-nt RNA substrate. For longer time-course reactions with the lsRNA substrates, timepoints were taken at 5, 15, 30, 45, 60, 90, 120, 180, 300 min and 24 h. Each timepoint was analyzed by denaturing PAGE with 8 M urea. RNA cleavage product bands were quantified using ImageJ software and the fraction cleaved was determined by the ratio of intensity between cleaved and total (uncleaved + cleaved) RNA for each reaction. The data was then fit to the one-phase exponential association equation, $Y = {Y}_{max}( {1 - {e}^{ - {k}_{obs}t}} )$, using GraphPad Prism 8 to obtain the first-order rate constant, *k*_obs_. Reactions with rationally designed (RD) ASOs were conducted as above, with the ASOs added in equimolar concentration to the 10–23 DNAzyme at 500 pM each. The fold increase in DNAzyme activity between lsRNA and sRNA transcripts was calculated as 100 – (*k*_obs lsRNA_/*k*_obs sRNA_) × 100, where *k*_obs lsRNA_ and *k*_obs sRNA_ represent the rate with the lsRNA and the matching sRNA, respectively. Similarly, the fold increase in DNAzyme activity with and without ASOs was calculated as 100 – (*I*_-ASO_/*I*_+ASO_) × 100, where *I*_-ASO_ and *I*_+ASO_ represent the intensity of the DNAzyme binding site cleavage bands within the in-line probing gel without and with ASOs.

### In-line probing

All in-line probing reactions were performed at room temperature for 48 h and contained ∼1 pM 5′-labeled ^32^P RNA in 10 μl of 1 × in-line reaction buffer (50 mM Tris–HCl pH 8.3, 20 mM MgCl_2_ and 100 mM KCl). In-line probing reactions with RD ASOs also contained 500 pM of each RD ASO. The reaction was quenched with 10 μl of 2 × colorless gel-loading solution (5 M urea and 0.75 mM EDTA pH 8.0). To analyze the nucleotide positions of the in-line probing reaction, a partial alkaline digestion was also performed with the radiolabeled RNA in 1 × Na_2_CO_3_ buffer (50 mM Na_2_CO_3_ pH 9.0 and 1 mM EDTA). The reaction mixture (10 μl) was incubated at 90°C for 6 min and quenched with 10 μl of 2 × colorless gel-loading solution. An RNase T1 digest was also performed which included ∼ 1 pM radiolabeled RNA in 10 μl of 1 × sodium citrate buffer (25 mM sodium citrate, pH 5.0), 10 units of RNase T1 (ThermoFisher scientific) and 3 μl of 2 × colorless gel-loading solution. The reaction was incubated at 65°C for 15 min, then quenched with 10 μl of 2 × colorless gel-loading solution. All reactions were analyzed by denaturing PAGE with 8 M urea, dried for 2 h at 65°C, exposed on an Amersham Biosciences storage phosphor screen overnight and imaged on an Amersham Typhoon 9200 scanner.

### Experimental ASO screen

Each experimental ASO screening reaction contained 500 pM experimental screening ASO (esASO), 500 pM 10–23 DNAzyme and ∼1 pM 5′-^32^P labeled lsRNA substrate in 10 μl of 1 × buffer 1. Reactions were heated for 1 min at 90°C and cooled for 10 min to allow the esASO and DNAzyme to hybridize to the lsRNA substrate. Reactions were initiated by addition of 1 × buffer 1 and incubated for 90 min or 24 h at room temperature. The esASO reactions were halted by addition of 10 μl of 2 × quenching buffer on ice and analyzed by denaturing PAGE with 8 M urea. The gels were exposed on an Amersham Biosciences storage phosphor screen for 2 h and imaged on an Amersham Typhoon 9200 scanner.

## Results and discussion

### Selection of eligible DNAzyme/lsRNA pairs for the study

Based on our earlier study in which 230 10–23 DNAzymes were screened for the most effective DNAzyme/target site combinations for cleaving the genomic RNA of SARS-CoV-2 (15), we selected five representative DNAzymes (dZ-1 to dZ-5) and three lsRNAs (lsRNA 1–3) in this study, with all sequences shown in Supplementary Table S1. lsRNA-1, lsRNA-2 and lsRNA-3, which contain 584, 566 and 831 nucleotides respectively, are three large RNA transcripts from the SARS-CoV-2 genome. Each 10–23 DNAzyme cleaves a different location within these transcripts, where dZ-1 and dZ-4 target two different sites within lsRNA-1 for cleavage, dZ-2 and dZ-5 target two different sites within lsRNA-2 for cleavage and dZ-3 targets one site within lsRNA-3 for cleavage (Supplementary Figure S1). All five DNAzymes were uniformly designed with substrate recognition arms of 15-nt on the 5′ end and 8-nt on the 3′ end (Supplementary Figure S2). The DNAzymes were placed in two categories according to the activity observed in our previous study: high activity (>20% cleavage observed with an lsRNA in a 10 min reaction) and low activity (<1% cleavage in a 10 min reaction). Specifically, dZ-4 and dZ-5 are high-activity DNAzymes while dZ-1, dZ-2 and dZ-3 are low-activity ones.

To observe the impact of RNA structure on catalytic activity, each of the five DNAzymes selected above were first assessed for percent cleavage (%Cleavage) at different time points in a 90-min reaction (Y_90_) against a pair of matching lsRNA (which represented structured RNA species) and 25-nt short RNA (sRNA, which represented unstructured or less structured RNA species). The data were used to derive cleavage rate constant (*k* observed or *k*_obs_) values. Comparing the *k*_obs_ and Y_90_ between the long (lsRNA) and short (sRNA) substrates provided a quantitative assessment of the impact of the RNA structure associated with the lsRNA on the accessibility of each targeted cleavage site within the lsRNA.

As expected, in the case of dZ-4 (Supplementary Figure S3) and dZ-5 (Supplementary Figure S4), which showed high activities in our previous screening study, the *k*_obs_ value for each DNAzyme against its sRNA substrate was only ∼2-fold higher than the matching lsRNA. These results can be interpreted as evidence that the cleavage sites targeted by these DNAzymes were in a relatively unstructured region of the lsRNA to which these DNAzymes had easy access. As a side note, we noticed that the rate of cleavage with dZ-5 for its short RNA (sRNA-5) substrate (*k*_obs_ = 0.17 min^−1^) was substantially (∼3-fold) higher than that observed for dZ-4 with its short RNA substrate sRNA-4 (*k*_obs_ = 0.056 min^−1^). We suspect that this rate difference may be due to intrinsic folding characteristics (59,60) of dZ-4, which can negatively impact recognition to the RNA substrate. Furthermore, since the nucleotide sequence surrounding the dZ-4 cleavage site within sRNA-4 is complementary to the substrate recognition arm sequence of dZ-4, sRNA-4 likely participates in a similar folding motif as dZ-4, further contributing to the lack of recognition efficiency between the DNAzyme and the sRNA substrate.

**Figure 1.**
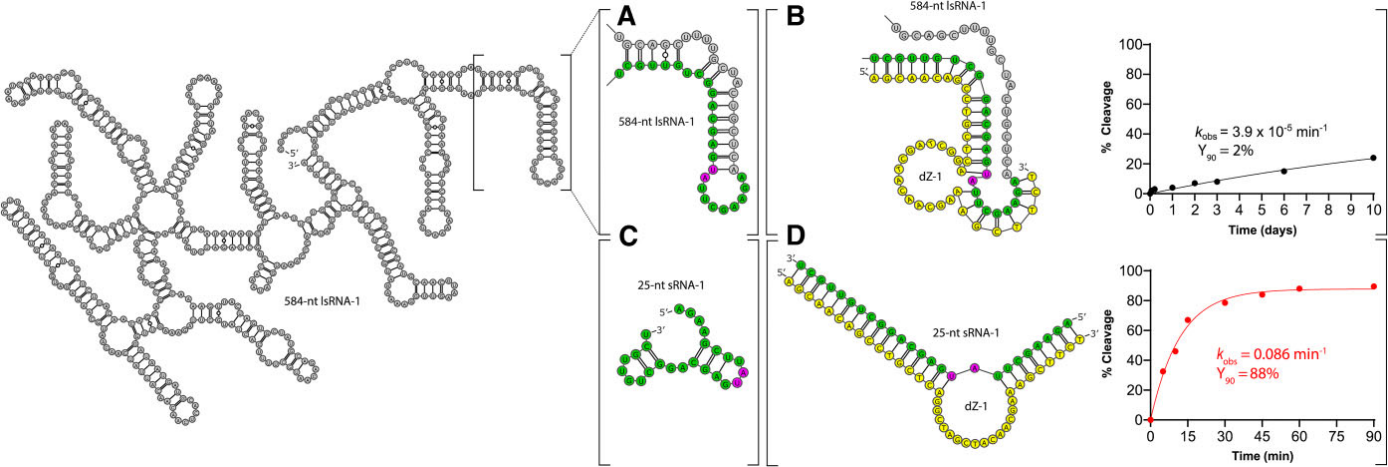
RNA cleavage activity comparison of a 10–23 DNAzyme, dZ-1, with a 584-nt RNA substrate, lsRNA-1 (**A**, **B**) and short 25-nt RNA substrate, sRNA-1 (**C**, **D**). The dZ-1 target site is shown in green for both lsRNA-1 (A) and sRNA (C) with A-U (diribonucleotides at cleavage site) shown in pink and dZ-1 is shown in yellow.

In stark contrast to the two DNAzyme systems discussed above, dZ-1, dZ-2 and dZ-3, which performed poorly in our previous screening assay, possessed relatively low *k*_obs_ and Y_90_ with their respective lsRNA substrates. However, catalytic activity was restored with each relevant sRNA, albeit with varying degrees of activity, which was likely due to intrinsic folding characteristics of the DNAzyme or its sRNA substrate, as mentioned above. We also note that even under fully optimized reaction conditions (25 mM Mg^2+^, 37°C, short RNA substrate), variation in activity was still observed between dZ-1, dZ-2 and dZ-3, highlighting the impact that binding arm sequence variation has on the degree of intrinsic folding (Supplementary Figure S5). Taking dZ-1 as an example, limited activity (*k*_obs_ = 3.9 × 10^−5^ min^−1^, Y_90_ = 2%) was observed with the lsRNA-1 substrate (Figure 1A & B), but with sRNA-1, it was able to cleave 88% after 90 min (Y_90_ = 88%) with a *k*_obs_ of 0.086 min^−1^ (Figure 1C, D). The over 2200-fold increase between the *k*_obs_ of the larger (lsRNA-1) substrate and shorter (sRNA-1) substrate clearly indicate that the targeted cleavage site was located within a region that could not be easily accessed by dZ-1, presumably due to its participation in the creation of a stable structure within the lsRNA, which greatly hindered the accessibility of dZ-1. Another interesting observation was the following: even though dZ-1 and dZ-4 were designed to target lsRNA-1 for cleavage, they possessed significantly different activities, with very limited catalytic activity observed for dZ-1 (*k*_obs_ = 3.9 × 10^−5^ min^−1^) compared to a *k*_obs_ of 0.027 min^−1^ for dZ-4, a ∼700-fold difference. For comparison, the *k*_obs_ values determined for the same two DNAzymes for sRNA-1 and sRNA-4 substrates respectively, differed by only ∼1.5-fold (0.086 min^−1^ for dZ-1 vs. 0.056 min^−1^ for dZ-4). This observation clearly indicates that both highly accessible and inaccessible target sites exist within the same lsRNA transcript, in this case, lsRNA-1.

### Improving DNAzyme accessibility to lsRNA with antisense oligonucleotides

Based on the data above, dZ-1, dZ-2 and dZ-3 were further investigated to determine if ASOs could be used to modify the secondary structure in the region of the lsRNA cleavage sites and thereby improve the accessibility and ultimately the catalytic activity of these 10–23 DNAzyme systems. As a first step, we used a ‘rationally designed’ ASO approach wherein 40-nt DNA sequences complementary to either the 3′ upstream (ASO A) or 5′ downstream (ASO B) region of the lsRNA cleavage site were designed, with a 3-nt to 5-nt gap between the ASO and DNAzyme binding arm regions on either side to facilitate conformational mobility. A length of 40-nt was chosen for two main reasons. First, since the cumulative 23-nt hybridization length of the three 10–23 DNAzyme substrate recognition arms were unable to effectively access lsRNA targets, 40-nt ASOs were hypothesized to better hybridize to these structured regions. Second, 40-nt ASOs are an average between the lengths of ASOs reported in similar studies, ranging from as low as 13-nt (51), up to 60-nt (55), therefore serving as a compromise for the inverse relationship between the ability to disrupt the RNA duplex of the target site and synthesis cost.

**Figure 2.**
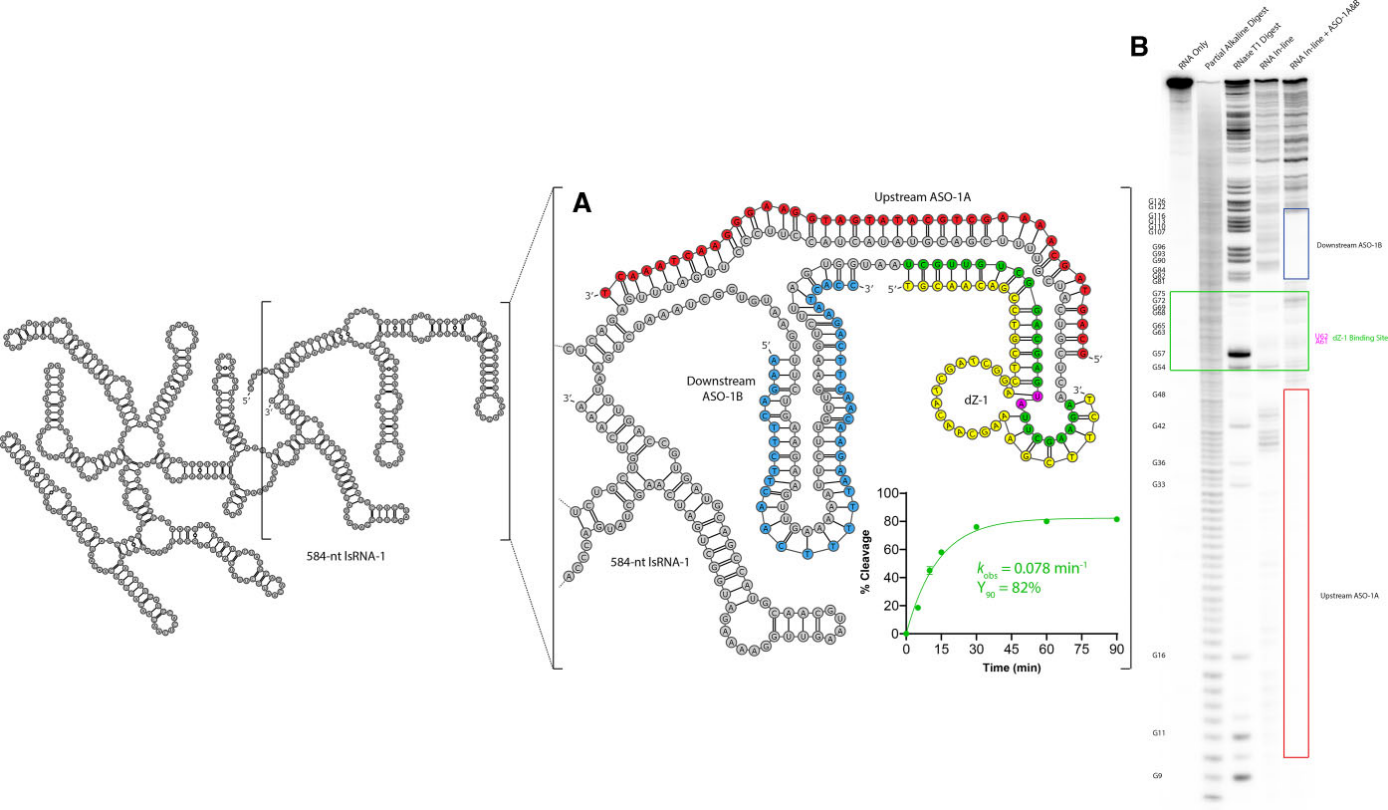
Improvement of dZ-1 activity and accessibility against lsRNA-1 with rationally designed ASO-1A and B. ASO-1A (red sequence) and B (blue sequence), both 40-nt in length, hybridized to upstream and downstream sequence elements of the dZ-1 binding site (shown in green) within lsRNA-1. Addition of ASO-1A and B restored dZ-1 activity, enabling dZ-1 to cleave 82% of lsRNA-1 after 90 min with a *k*_obs_ of 0.078 min^−1^ (**A**). Addition of ASO-1A and B to lsRNA-1 also increased the accessibility of dZ-1 by reducing RNA secondary structure of the dZ-1 binding site, as shown by in-line probing analysis (**B**). The in-line probing reaction was conducted using lsRNA-1 without ASO-1A or B (‘RNA in-line’) and with ASO-1A and B (‘RNA in-line + ASO-1A&B’).

We first measured the effect of using a combination of both upstream and downstream rationally designed ASOs on DNAzyme activity with lsRNA substrates using kinetic analysis, as described above. For dZ-1, limited activity was observed without ASOs (*k*_obs_ = 3.9 × 10^−5^ min^−1^, Y_90_ = 2%). However, when both ASO-1A and B were added, dZ-1 cleavage activity with lsRNA-1 was restored, cleaving 82% of lsRNA-1 after 90 min (Y_90_ = 82%) with a *k*_obs_ of 0.073 min^−1^ (Figure 2A). This corresponds to an increase of 2000-fold in *k*_obs_ with ASO-1A and B, relative to the *k*_obs_ obtained without ASOs. Additionally, the rate of lsRNA-1 cleavage by dZ-1 with ASO-1A and B was only 15% lower than the dZ-1 cleavage of the sRNA-1 substrate indicating a significant reduction in the structure of the dZ-1 binding site within lsRNA-1.

ASO-1A and B were also tested separately to determine their effectiveness at improving dZ-1 accessibility to lsRNA-1 compared to their combined application. ASO-1A and B produced similar improvements to each other in lsRNA-1 cleavage activity by dZ-1 when added separately, with *k*_obs_ = 0.011 min^−1^ and *k*_obs_ = 0.0086 min^−1^ for ASO-1A and B, respectively, which was 7-fold and 9-fold slower than the *k*_obs_ = 0.078 min^−1^ obtained when both ASO-1A and B were added together, indicating that both ASO-1A and B are required for optimal lsRNA-1 cleavage by dZ-1 (Supplementary Figure S6).

For a deeper insight into the impact of ASO-1A and B on lsRNA-1 structure in the region of the cleavage site, we also performed an in-line probing experiment (61) with lsRNA-1 and compared the intensities of the dZ-1 binding site with and without the ASO-1A and B combination (Figure 2B). Areas with higher intensity correlate to an increase in the ‘in-line’ conformation of the RNA phosphodiester backbone, increasing the rate of transesterification and indicating reduced structure in this region. The combined addition of ASO-1A and B increased lsRNA-1 transesterification of the dZ-1 binding site by 51% relative to the native lsRNA-1 without ASOs, consistent with a reduction in the degree of secondary structure.

**Figure 3.**
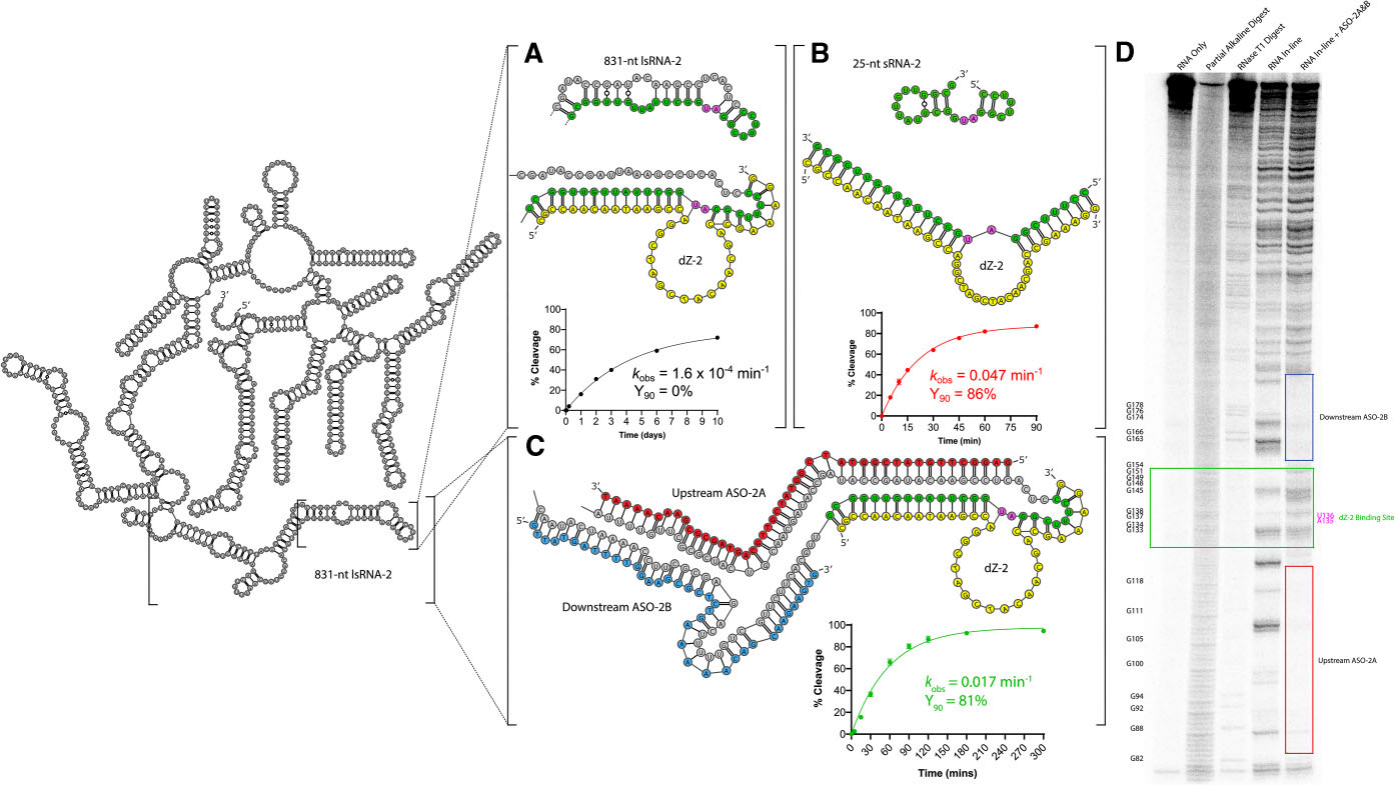
RNA cleavage activity comparison of a 10–23 DNAzyme, dZ-2 with the 831-nt lsRNA-2 (**A**) and short 25-nt sRNA-2 (**B**), followed by optimization with rationally designed ASOs (**C**). The dZ-2 target site is shown in green for both lsRNA-2 (A) and sRNA-2 (B) substrates with AU (diribonucleotides at the cleavage site) shown in pink. dZ-2 (shown in yellow) has limited activity with lsRNA-2 (*k*_obs_ = 1.6 × 10^−4^ min^−1^) (A), but cleaves sRNA-2 with a *k*_obs_ of 0.047 min^−1^ and Y_90_ of 86% (B). ASO-2A and B (shown in red and blue, respectively), 40-nt in length, hybridized to upstream and downstream sequence elements of the dZ-2 binding site within lsRNA-2 and restored dZ-2 activity, cleaving 81% of lsRNA-2 after 90 min with a *k*_obs_ of 0.017 min^−1^ (**C**). Addition of both ASO-2A and B to lsRNA-2 also increased the accessibility of the dZ-2 by reducing RNA secondary structure of the dZ-2 binding site as shown by in-line probing analysis (**D**).

Next, we tested the rationally designed ASO approach with dZ-2, which possessed limited activity for the lsRNA-2 substrate (*k*_obs_ = 1.6 × 10^−4^ min^−1^, Y_90_ = 2%) (Figure 3A), but regained activity with the shorter sRNA-2 substrate, cleaving 86% after 90 min (Y_90_ = 86%) with a *k*_obs_ of 0.047 min^−1^ (Figure 3B). When both ASO-2A and B were added to lsRNA-2, we observed 81% cleavage by dZ-2 after 90 min (Y_90_ = 81%) with a *k*_obs_ of 0.017 min^−1^ (Figure 3C), corresponding to a 106-fold increase in *k*_obs_ relative to the same reaction without ASOs, though this was 4.3-fold slower than the rate of lsRNA-1 cleavage by dZ-1 with ASO-1A and B. Moreover, the rate of lsRNA-2 cleavage by dZ-2 with both ASO-2A and B was 23% that of sRNA-2, whereas dZ-1 with ASOs reached 85% of the rate observed with sRNA-1, suggesting that the ASOs were not able to fully open the structure of lsRNA-2 to the same degree as lsRNA-1.

As with dZ-1, the activity of dZ-2 was tested with each rationally designed ASO independently. When only the upstream ASO-2A was added, dZ-2 cleaved lsRNA-2 with a *k*_obs_ = 0.0082 min^−1^ and Y_90_ = 21%, and with only the downstream ASO-2B, 25% was cleaved after 90 min (Y_90_ = 25%) with a *k*_obs_ = 0.0018 min^−1^, corresponding to a 2-fold and 9-fold decrease in *k*_obs_ respectively, relative to the *k*_obs_ = 0.017 min^−1^ obtained when both ASO-2A and B were added together (Supplementary Figure S6). These results show that for both dZ-1 and dZ-2, the greatest improvements in lsRNA cleavage activity required binding of both rationally designed ASO-A and B. The increase in catalytic performance with the combination of both ASOs was further corroborated by in-line probing analysis, which showed an increase in RNA transesterification by 39% with lsRNA-2 for the dZ-2 binding site, compared to the no ASO control (Figure 3D). This is lower than the value obtained for lsRNA-1 with ASO-1A and B (51% improvement), again suggesting that lsRNA-2 accessibility was not improved to the same extent as lsRNA-1, though there was an overall reduction in structural elements in the DNAzyme binding region provided by the ASOs, resulting in greater DNAzyme accessibility.

**Figure 4.**
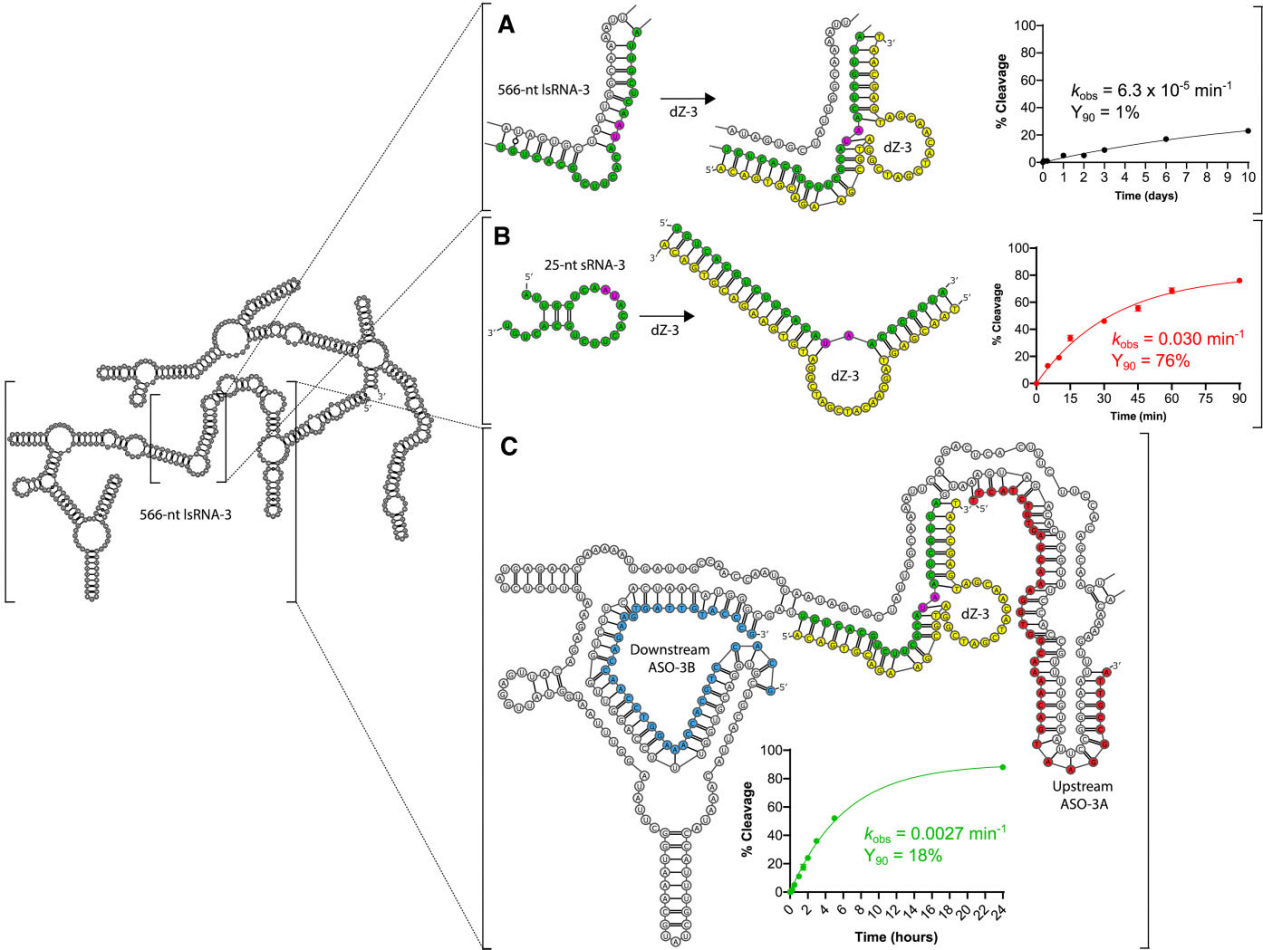
RNA cleavage activity comparison of a 10–23 DNAzyme, dZ-3 with 566-nt lsRNA-3 (**A**) and sRNA-3 (**B**), followed by optimization with rationally designed ASOs (**C**). The dZ-3 target site is shown in green for both lsRNA-3 (A) and sRNA-3 (B) with AU (the diribonucleotides at the cleavage site) shown in pink. dZ-3 (shown in yellow) is poorly active with lsRNA-3 (*k*_obs_ = 6.3 × 10^−5^ min^−1^) (A), but cleaves sRNA-3 with a *k*_obs_ of 0.030 min^−1^ and Y_90_ of 76% (B). ASO-3A and B (shown in red and blue, respectively), 40-nt in length, hybridized to upstream and downstream sequence elements of the dZ-3 binding site and cleaved 18% of lsRNA-3 after 90 mins with a *k*_obs_ of 0.0027 min^−1^ (**C**).

For the last DNAzyme, dZ-3, a similar trend was observed compared to dZ-1 and dZ-2, where dZ-3 activity against lsRNA-3 was poor (*k*_obs_ = 6.3 × 10^−5^ min^−1^, Y_90_ = 2%) (Figure 4A), while with sRNA-3, it was able to cleave 76% within 90 min (Y_90_ = 76%) with a *k*_obs_ of 0.030 min^−1^ (Figure 4B). However in contrast to dZ-1 and dZ-2, when both ASO-3A and B were added, we observed only 18% of lsRNA-3 cleaved by dZ-3 after 90 min (Y_90_ = 18%) with a *k*_obs_ of 0.0027 min^−1^, which corresponded to a 43-fold increase in the rate of lsRNA-3 cleavage by dZ-3 relative to the rate (*k*_obs_) of the same reaction without ASOs. Furthermore, the rate of lsRNA-3 cleavage by dZ-3 with ASO-3A and B (*k*_obs_ = 0.0027 min^−1^) was 6.4-fold slower compared to the dZ-2/lsRNA-2 system (*k*_obs_ = 0.017 min^−1^) and 27-fold slower than the dZ-1/lsRNA-1 system (*k*_obs_ = 0.073 min^−1^). Unlike these latter two systems, for the dZ-3/lsRNA-3 system only ASO-3B was necessary to achieve sufficient lsRNA-3 cleavage by dZ-3, with a *k*_obs_ = 0.0025 min^−1^ and Y_90_ = 15%, which was similar to the *k*_obs_ = 0.0027 min^−1^ and Y_90_ = 18% with both ASO-3A and B present. The upstream ASO-3A, by contrast, provided no improvement in lsRNA-3 cleavage by dZ-3, achieving a linear *k*_obs_ = 2.1 × 10^−5^ min^−1^ and Y_90_ = 1%, which was 119-fold slower compared to the rate of dZ-3 cleavage of lsRNA-3 with only ASO-3B (*k*_obs_ = 0.0025 min^−1^) (Supplementary Figure S6). The relatively poor lsRNA-3 cleavage activity by dZ-3, even with ASO-3A and B, suggests that the binding site remained partially inaccessible, potentially due to either retention of local secondary structure around the cleavage site or additional impact from tertiary structures in lsRNA and that for some systems, only one ASO is necessary to achieve sufficient lsRNA cleavage activity.

To gain greater insight into the variability that ASOs elicit in modulation of lsRNA structure, secondary structures of the lsRNA substrates were generated using a minimum free energy (MFE) approach. We note, however, that these predicted structures are only one of several low energy structures, and that it is likely that only a subpopulation of lsRNA may adopt the MFE structure shown. In the case of dZ-1 (Figure 2) and dZ-2 (Figure 3), the binding site for the upstream ASO A and the binding site for the DNAzyme partially overlapped, targeting complementary RNA nucleotides within the same duplex structure in the lsRNA substrates. Conversely for dZ-3, there was no overlap of ASO-3A or ASO-3B with the binding site of dZ-3 on the lsRNA-3 transcript (Figure 4). This may suggest that the combined hybridization of both strands of the RNA duplex for the dZ-1/lsRNA-1 and dZ-2/lsRNA-2 systems by ASO A, improves DNAzyme accessibility through a cooperative binding mechanism, where hybridization to one strand of the RNA duplex improves hybridization to the complementary strand. Conversely, for dZ-3/lsRNA-3, hybridization to only one strand of the RNA duplex may not elicit this effect and leave the complementary strand in a single stranded conformation, with the possibility of competing with ASO-3A, B and dZ-3 hybridization. In addition, there may be other structural elements within the lsRNA-3 substrate, which exist outside of the ASO-3A and B binding sites, that limit dZ-3 accessibility since lsRNA is likely to form tertiary structures that may limit accessibility to certain target sites within the molecule.

### Experimental antisense oligonucleotide screening approach to identify additional lsRNA structure space to improve DNAzyme accessibility

The rationally designed ASO approach was able to modify the lsRNA secondary structure in a manner that significantly improved accessibility and activity for dZ-1 and dZ-2 for their respective lsRNA substrates, achieving fold improvements in *k*_obs_ of 2000-fold and 106-fold respectively compared to the *k*_obs_ without ASOs. Although this approach also significantly improved dZ-3 activity for lsRNA-3, it was less effective compared to dZ-1 and dZ-2 for their respective lsRNA substrates, with a 43-fold improvement in *k*_obs_ compared to the no ASO reaction, which was 27-fold and 6.4-fold slower than dZ-1 and dZ-2 respectively.

It is known that larger RNA molecules readily form tertiary structures in solution (62), and as a result, it was hypothesized that tertiary structures within lsRNA-3 were potentially hindering accessibility of dZ-3 to the cleavage site. In previous work, ASO binding to distant regions of transfer-messenger RNA was shown to impact the RNA tertiary structure in a manner that improved the hybridization efficiency of a DNA probe (47). From this, we devised an experimental ASO screening approach to identify more distant regions from the dZ-3 binding site within lsRNA-3 that might be impacting dZ-3 accessibility, instead of only immediately upstream or downstream of the dZ-3 binding site. These experimental screening ASOs (esASOs) would theoretically disrupt any tertiary interactions within lsRNA-3 that were inhibiting dZ-3 accessibility and thus improve its activity.

**Figure 5.**
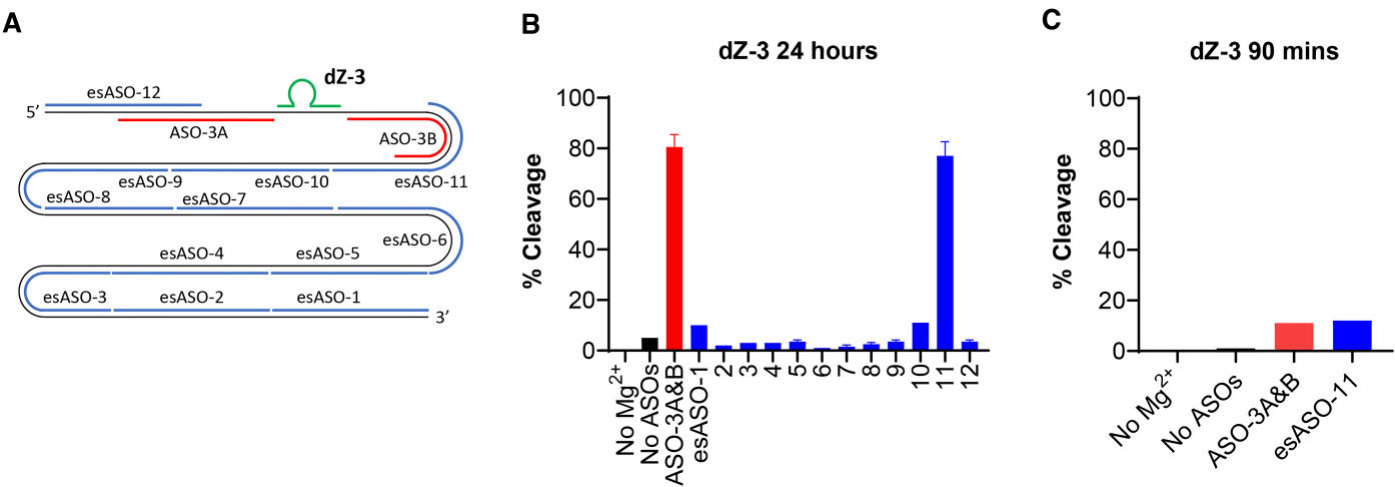
Experimental ASO screen for dZ-3 with experimental screening ASO (esASO) binding sites within lsRNA-3. Each screening reaction contained equimolar amounts of dZ-3 (green) and 40-nt esASO (blue) starting with esASO-1 from the 3′-end of lsRNA-3, with 12 esASOs total needed to cover the entire sequence space of lsRNA-3 (**A**). After the 24 h reaction, esASO-11 was found to elicit the most significant enhancment in dZ-3 cleavage for lsRNA-3 out of the 12 esASOs tested, but was not more effective than ASO-3A and B (**B**). The same trend was observed after 90 min (**C**).

To probe regions of the lsRNA-3 substrate for tertiary effects on dZ-3 activity, 40-nt long esASOs were designed to cover the entire lsRNA-3 sequence space, with a total of 12 esASOs tested (Figure 5A, Supplementary Figure S7). The 12 esASOs hybridized to regions upstream and downstream of the dZ-3 binding site for lsRNA-3, which were not identical to the binding sites for ASO-3A or B. It is important to note that each screening reaction used a single esASO rather than a combination of upstream and downstream ASOs as with ASO-3A and B. Furthermore, as it was unclear how large an impact the individual esASOs would have on dZ-3 activity, a longer reaction time was necessary to accurately measure the variation in cleavage activity for each esASO reaction, and thus, a single timepoint was taken at 24 h.

The results from the esASO screen showed that the highest %Cleavage for dZ-3 was obtained for esASO-11, which hybridized to a region within lsRNA-3 that was immediately downstream of the dZ-3 binding site, overlapping with twelve nucleotides of the initial downstream ASO-3B (Figure 5B). However, although esASO-11 was found to elicit the greatest improvement in dZ-3 accessibility from the screen, it provided no significant enhancement in lsRNA-3 cleavage compared to the combined application of ASO-3A and B, achieving the same *k*_obs_ of 0.0027 min^−1^ and a similar fraction of lsRNA-3 cleaved, with 17% (Y_90_ = 17%) and 18% (Y_90_ = 18%) respectively after 90 min (Figure 5C, Supplementary Figure S8). Importantly, we found that more distant esASOs, aside from esASO-11, produced statistically insignificant improvements in dZ-3 activity, indicating that tertiary interactions between more distant regions and the dZ-3 binding site within lsRNA-3 likely had limited impact on accessibility compared to secondary structural interactions. This trend is further corroborated by esASO screens for dZ-1 and dZ-2, which both showed that the top esASOs hybridized to structured regions close to their respective DNAzyme binding sites and overlapped with either the upstream or downstream rationally designed ASO binding site (Supplementary Figure S9). Since neither the rationally designed ASOs (ASO-3A & B) nor the esASO screening approach significantly improved dZ-3 activity for lsRNA-3, we explored two distinct strategies to achieve similar fold improvements in lsRNA accessibility for dZ-3 as compared to dZ-1 and dZ-2 for their respective lsRNA substrates.

Since the combined application of rationally designed ASOs improved dZ-1 and dZ-2 activity for lsRNA-1 and lsRNA-2 more than with each ASO individually, the first strategy involved testing whether the combined application of top esASOs from the experimental screen could enable dZ-3 to achieve a similar fold increase in activity with lsRNA-3. We first tested a combination of esASO-10 and esASO-11, which enabled dZ-3 to cleave 42% of lsRNA-3 after 90 min (Y_90_ of 42%), with a *k*_obs_ of 0.0070 min^−1^, corresponding to a 2.6-fold improvement in *k*_obs_ compared to using ASO-3A and B together (Supplementary Figure S8). We then tested esASO-1 (which hybridized to the 3′-terminus of lsRNA-3) and esASO-11 together and observed a similar 41% of lsRNA-3 cleavage by dZ-3 after 90 min (Y_90_ = 41%), with a *k*_obs_ of 0.0078 min^−1^ (2.9-fold improvement relative to ASO-3A and B together) (Supplementary Figure S8). Both of these methods, which used ASOs that were identified from the esASO screen, improved the rate (*k*_obs_) and fraction of lsRNA-3 cleaved (Y_90_) by dZ-3 more than with the rationally designed ASO-3A and B.

The second strategy involved the extension of a single ASO from 40-nt to 60-nt, to both improve the hybridization efficiency to lsRNA-3 and reduce additional secondary structure elements that may inhibit dZ-3 accessibility. Since the experimental screen showed that secondary structural interactions had a more significant impact on dZ-3 binding to lsRNA-3 compared to more distal tertiary interactions, the rationally designed downstream ASO-3B was the first logical candidate for extension. However, when a 20-nt extension to the 3′-end of ASO-3B was tested, only 8% of lsRNA-3 was cleaved by dZ-3 after 90 min (Y_90_ = 8%) with a *k*_obs_ of 9.1 × 10^−4^ min^−1^, which was 2.7-fold less effective compared to the rate of lsRNA-3 cleaved by dZ-3 with the original 40-nt ASO-3B alone, with a *k*_obs_ = 0.0025 min^−1^ and Y_90_ = 15% (Supplementary Figures S6, S10).

**Figure 6.**
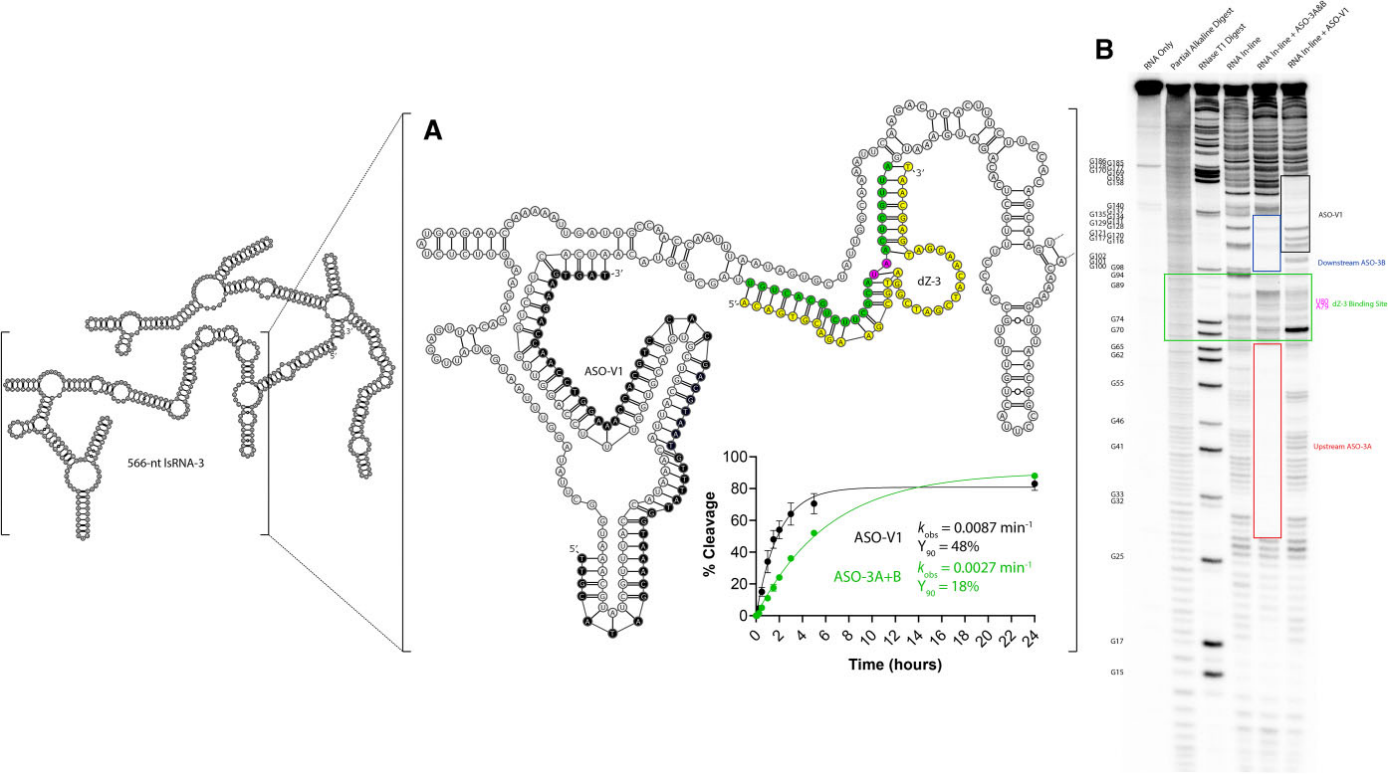
Improvement of dZ-3 (yellow) activity and accessibility for lsRNA-3 with variant 1 of esASO-11 (ASO-V1, black), which was identified through an experimental ASO screen and possesses an additional 20-nt to its 3′ terminus (**A**). ASO-V1 increased the rate (*k*_obs_) and fraction cleaved (Y_90_) of lsRNA-3 by dZ-3 (black curve) compared to the same reaction with rationally designed ASO3-A and B (green curve). The addition of ASO-V1 also increased the accessibility of the dZ-3 binding site within lsRNA-3 by reducing RNA secondary structure of the binding site (green box) as shown by in-line probing analysis (**B**).

The next candidate was esASO-11, which showed nearly an identical impact in dZ-3 accessibility to lsRNA-3 compared to ASO-3B and was located further downstream on the lsRNA-3 transcript, overlapping with 12 nucleotides of the ASO-3B binding site as mentioned previously. A total of three separate extended esASO-11 variants, now 60-nt in length, were designed and tested to improve dZ-3 accessibility to lsRNA-3 (Supplementary Figure S10). Variant 1 possessed an additional 20 nucleotides to the 3′ end of esASO-11 (termed ASO-V1) and was found to facilitate the greatest improvement in dZ-3 activity, cleaving 48% of lsRNA-3 after 90 min (Y_90_ = 48%) with a *k*_obs_ of 0.0087 min^−1^ (3.2-fold faster *k*_obs_ compared to the rate of lsRNA-3 cleavage with ASO-3A and B together (*k*_obs_ = 0.0027 min^−1^)) (Figure 6A). When compared to the rate of dZ-3 with lsRNA-3 (*k*_obs_ = 3.9 × 10^−5^ min^−1^), ASO-V1 elicited a 138-fold improvement in the rate (*k*_obs_) of lsRNA-3 cleavage by dZ-3, which was greater than any other single ASO or ASO combination for dZ-3. To gain a mechanistic insight into how ASO-V1 improved dZ-3 accessibility to lsRNA/-3, an in-line probing experiment was performed. The results showed an increase in RNA transesterification of 56% with lsRNA-3 for the dZ-3 binding site when ASO-V1 was added, compared to only 45% with the rationally designed ASO-3A and B, corresponding to a greater reduction in secondary structure formation that was consistent with the observed rate improvement (Figure 6B).

The significant improvement with ASO-V1 highlights the impact that lsRNA structure has on accessibility with antisense agents including both DNAzymes and non-catalytic DNA oligonucleotides like ASOs. In the case with esASO-11, it is likely that a length of 40-nt was not sufficient to fully linearize the dZ-3 binding site because of significant structural formation within lsRNA-3. Thus in this case, extending the 3′-end of esASO-11 by an additional 20 nucleotides to a final size of 60 nucleotides was necessary to improve dZ-3 accessibility. Looking at the in-line probing reaction for ASO-V1, although it reduced the structure of the dZ-3 binding site within lsRNA-3, it was unable to completely hybridize to the lsRNA-3 substrate, especially towards its 3′ terminus. It is also important to note that while longer ASOs may elicit a greater improvement in DNAzyme accessibility compared to shorter ASOs, the trade-off between DNA synthesis cost and lsRNA cleavage activity must be considered for practical applications.

In summary, testing five 10–23 DNAzymes against lsRNA substrates revealed three DNAzymes that were unable to access their structured target sites within lsRNA molecules of biological origin. Rationally designed ASOs located just upstream or downstream of the DNAzyme cleavage site improved DNAzyme accessibility to these target sites and restored catalytic activity, though the degree of improvement varied for different lsRNA systems. We also designed an ASO screening method to probe for potential inhibitory tertiary interactions elsewhere within the lsRNA sequence space and found that localized structures have a greater impact on DNAzyme accessibility. However, extension of a top ASO from the screen provided a significant improvement in DNAzyme accessibility compared to the rationally designed ASOs in one lsRNA system. Overall, this study shows that rationally designed ASOs enable previously inaccessible target sites within lsRNA molecules to be accessible to 10–23 DNAzymes. We also show that experimental ASO screening can be used to guide the design of new ASOs that improve 10–23 DNAzyme accessibility. Taken together, these strategies can improve accessibility of other biological catalysts to structured target sites within lsRNA substrates.

## Supplementary Material

gkae778_Supplemental_File

## Data Availability

The data underlying this article are available in the article and in its online supplementary material.
