# Supplementary material for: Making target sites in large structured RNAs accessible to RNA-cleaving DNAzymes through hybridization with synthetic DNA oligonucleotides
Source: Nucleic Acids Res. 2024 Sep 9;52(18):11177–87. doi: 10.1093/nar/gkae778 (PMC11472044; doi:10.1093/nar/gkae778)
Supplement: gkae778_Supplemental_File [file gkae778_supplemental_file.docx]

**Making Target Sites in Large Structured RNAs Accessible to RNA-cleaving DNAzymes With Antisense DNA Oligonucleotides**

Supplementary Information

Table S1. Sequences of all relevant DNA and RNA oligonucleotides. The 10-23 DNAzyme catalytic core is bolded and underlined, and the AU cleavage site for each 25-nt RNA substrate is bolded.

| Oligonucleotide | Length (nucleotides) | Sequence (5’ 🡪 3’) |
| --- | --- | --- |
| **RNA** |  |  |
| sRNA-1 (dZ-1 RNA substrate) | 25 | AGAAGCUU**AU**GAGCAGGCUGUUGCU |
| sRNA-2 (dZ-2 RNA substrate) | 25 | CCUUUCGG**AU**GGCUUAUUGUUGGCG |
| sRNA-3 (dZ-3 RNA substrate) | 25 | AUUGCUCA**AU**ACACUUCUGCACUGU |
| sRNA-4 (dZ-4 RNA substrate) | 25 | UAUGGACA**AU**UCACCUAAUUUAGCA |
| sRNA-5 (dZ-5 RNA substrate) | 25 | AAAAACCC**AU**UACUUUAUGAUGCCA |
| lsRNA-1 from SARS-CoV-2 *NSP8* 12098/12679 | 584 | GGGCCUCAGAGUUUAGUUCCCUUCCAUCAUAUGCAGCUUUUGCUACUGCUCAAGAAGCUUAUGAGCAGGCUGUUGCUAAUGGUGAUUCUGAAGUUGUUCUUAAAAAGUUGAAGAAGUCUUUGAAUGUGGCUAAAUCUGAAUUUGACCGUGAUGCAGCCAUGCAACGUAAGUUGGAAAAGAUGGCUGAUCAAGCUAUGACCCAAAUGUAUAAACAGGCUAGAUCUGAGGACAAGAGGGCAAAAGUUACUAGUGCUAUGCAGACAAUGCUUUUCACUAUGCUUAGAAAGUUGGAUAAUGAUGCACUCAACAACAUUAUCAACAAUGCAAGAGAUGGUUGUGUUCCCUUGAACAUAAUACCUCUUACAACAGCAGCCAAACUAAUGGUUGUCAUACCAGACUAUAACACAUAUAAAAAUACGUGUGAUGGUACAACAUUUACUUAUGCAUCAGCAUUGUGGGAAAUCCAACAGGUUGUAGAUGCAGAUAGUAAAAUUGUUCAACUUAGUGAAAUUAGUAUGGACAAUUCACCUAAUUUAGCAUGGCCUCUUAUUGUAACAGCUUUAAGGGCCAAUUCUGCUGUCAAA |
| lsRNA-2 from SARS-CoV-2 *ORF3a* 25393/26220 | 831 | GGGAUGGAUUUGUUUAUGAGAAUCUUCACAAUUGGAACUGUAACUUUGAAGCAAGGUGAAAUCAAGGAUGCUACUCCUUCAGAUUUUGUUCGCGCUACUGCAACGAUACCGAUACAAGCCUCACUCCCUUUCGGAUGGCUUAUUGUUGGCGUUGCACUUCUUGCUGUUUUUCAGAGCGCUUCCAAAAUCAUAACCCUCAAAAAGAGAUGGCAACUAGCACUCUCCAAGGGUGUUCACUUUGUUUGCAACUUGCUGUUGUUGUUUGUAACAGUUUACUCACACCUUUUGCUCGUUGCUGCUGGCCUUGAAGCCCCUUUUCUCUAUCUUUAUGCUUUAGUCUACUUCUUGCAGAGUAUAAACUUUGUAAGAAUAAUAAUGAGGCUUUGGCUUUGCUGGAAAUGCCGUUCCAAAAACCCAUUACUUUAUGAUGCCAACUAUUUUCUUUGCUGGCAUACUAAUUGUUACGACUAUUGUAUACCUUACAAUAGUGUAACUUCUUCAAUUGUCAUUACUUCAGGUGAUGGCACAACAAGUCCUAUUUCUGAACAUGACUACCAGAUUGGUGGUUAUACUGAAAAAUGGGAAUCUGGAGUAAAAGACUGUGUUGUAUUACACAGUUACUUCACUUCAGACUAUUACCAGCUGUACUCAACUCAAUUGAGUACAGACACUGGUGUUGAACAUGUUACCUUCUUCAUCUACAAUAAAAUUGUUGAUGAGCCUGAAGAACAUGUCCAAAUUCACACAAUCGACGGUUCAUCCGGAGUUGUUAAUCCAGUAAUGGAACCAAUUUAUGAUGAACCGACGACGACUACUAGCGUGCCUUUGUAA |
| lsRNA-3 from SARS-CoV-2 Spike 24108/24665 | 566 | GGGUUUCCCCAUUUGUGCACAAAAGUUUAACGGCCUUACUGUUUUGCCACCUUUGCUCACAGAUGAAAUGAUUGCUCAAUACACUUCUGCACUGUUAGCGGGUACAAUCACUUCUGGUUGGACCUUUGGUGCAGGUGCUGCAUUACAAAUACCAUUUGCUAUGCAAAUGGCUUAUAGGUUUAAUGGUAUUGGAGUUACACAGAAUGUUCUCUAUGAGAACCAAAAAUUGAUUGCCAACCAAUUUAAUAGUGCUAUUGGCAAAAUUCAAGACUCACUUUCUUCCACAGCAAGUGCACUUGGAAAACUUCAAGAUGUGGUCAACCAAAAUGCACAAGCUUUAAACACGCUUGUUAAACAACUUAGCUCCAAUUUUGGUGCAAUUUCAAGUGUUUUAAAUGAUAUCCUUUCACGUCUUGACAAAGUUGAGGCUGAAGUGCAAAUUGAUAGGUUGATCACAGGCAGACUUCAAAGUUUGCAGACAUAUGUGACUCAACAAUUAAUUAGAGCUGCAGAAAUCAGAGCUUCUGCUAAUCUUGCUGCUACUAAAAUGUCAGAGUGUGUACUUG |
| **DNA** |  |  |
| dZ-1 (dZ12156a) | 39 | AGCAACAGCCTGCTC**AGGCTAGCTACAACG**AAAGCTTCT |
| dZ-2 (dZ25524a) | 39 | CGCCAACAATAAGCC**AGGCTAGCTACAACG**ACCGAAAGG |
| dZ-3 (dZ24178a) | 39 | ACAGTGCAGAAGTGT**AGGCTAGCTACAACG**ATGAGCAAT |
| dZ-4 (dZ12618a) | 39 | TGCTAAATTAGGTGA**AGGCTAGCTACAACG**ATGTCCATA |
| dZ-5 (dZ25806a) | 39 | TGGCATCATAAAGTA**AGGCTAGCTACAACG**AGGGTTTTT |
| ASO-1A | 40 | GCAGTAGCAAAAGCTGCATATGATGGAAGGGAACTAAACT |
| ASO-1B | 40 | AAGACTTCTTCAACTTTTTAAGAACAACTTCAGAATCACC |
| ASO-2A | 40 | GAGGCTTGTATCGGTATCGTTGCAGTAGCGCGAACAAAAT |
| ASO-2B | 40 | GTTATGATTTTGGAAGCGCTCTGAAAAACAGCAAGAAGTG |
| ASO-3A | 40 | TTCATCTGTGAGCAAAGGTGGCAAAACAGTAAGGCCGTTA |
| ASO-3B | 40 | GCACCTGCACCAAAGGTCCAACCAGAAGTGATTGTACCCG |
| esASO-11 variant 1 (ASO-V1) | 60 | TTGCATAGCAAATGGTATTTGTAATGCAGCACCTGCACCAAAGGTCCAACCAGAAGTGAT |
| esASO-11 variant 2 | 60 | CCATTAAACCTATAAGCCATTTGCATAGCAAATGGTATTTGTAATGCAGCACCTGCACCA |
| esASO-11 variant 3 | 60 | TATAAGCCATTTGCATAGCAAATGGTATTTGTAATGCAGCACCTGCACCAAAGGTCCAAC |
| dZ-1 esASO-1 | 40 | TTTGACAGCAGAATTGGCCCTTAAAGCTGTTACAATAAGA |
| dZ-1 esASO-2 | 40 | GGCCATGCTAAATTAGGTGAATTGTCCATACTAATTTCAC |
| dZ-1 esASO-3 | 40 | TAAGTTGAACAATTTTACTATCTGCATCTACAACCTGTTG |
| dZ-1 esASO-4 | 40 | GATTTCCCACAATGCTGATGCATAAGTAAATGTTGTACCA |
| dZ-1 esASO-5 | 40 | TCACACGTATTTTTATATGTGTTATAGTCTGGTATGACAA |
| dZ-1 esASO-6 | 40 | CCATTAGTTTGGCTGCTGTTGTAAGAGGTATTATGTTCAA |
| dZ-1 esASO-7 | 40 | GGGAACACAACCATCTCTTGCATTGTTGATAATGTTGTTG |
| dZ-1 esASO-8 | 40 | AGTGCATCATTATCCAACTTTCTAAGCATAGTGAAAAGCA |
| dZ-1 esASO-9 | 40 | TTGTCTGCATAGCACTAGTAACTTTTGCCCTCTTGTCCTC |
| dZ-1 esASO-10 | 40 | AGATCTAGCCTGTTTATACATTTGGGTCATAGCTTGATCA |
| dZ-1 esASO-11 | 40 | GCCATCTTTTCCAACTTACGTTGCATGGCTGCATCACGGT |
| dZ-1 esASO-12 | 40 | CAAATTCAGATTTAGCCACATTCAAAGACTTCTTCAACTT |
| dZ-1 esASO-13 | 40 | AAAGCTGCATATGATGGAAGGGAACTAAACTCTGAGGCCC |
| dZ-2 esASO-1 | 40 | TTACAAAGGCACGCTAGTAGTCGTCGTCGGTTCATCATAA |
| dZ-2 esASO-2 | 40 | ATTGGTTCCATTACTGGATTAACAACTCCGGATGAACCGT |
| dZ-2 esASO-3 | 40 | CGATTGTGTGAATTTGGACATGTTCTTCAGGCTCATCAAC |
| dZ-2 esASO-4 | 40 | AATTTTATTGTAGATGAAGAAGGTAACATGTTCAACACCA |
| dZ-2 esASO-5 | 40 | GTGTCTGTACTCAATTGAGTTGAGTACAGCTGGTAATAGT |
| dZ-2 esASO-6 | 40 | CTGAAGTGAAGTAACTGTGTAATACAACACAGTCTTTTAC |
| dZ-2 esASO-7 | 40 | TCCAGATTCCCATTTTTCAGTATAACCACCAATCTGGTAG |
| dZ-2 esASO-8 | 40 | TCATGTTCAGAAATAGGACTTGTTGTGCCATCACCTGAAG |
| dZ-2 esASO-9 | 40 | TAATGACAATTGAAGAAGTTACACTATTGTAAGGTATACA |
| dZ-2 esASO-10 | 40 | ATAGTCGTAACAATTAGTATGCCAGCAAAGAAAATAGTTG |
| dZ-2 esASO-11 | 40 | GCATCATAAAGTAATGGGTTTTTGGAACGGCATTTCCAGC |
| dZ-2 esASO-12 | 40 | AAAGCCAAAGCCTCATTATTATTCTTACAAAGTTTATACT |
| dZ-2 esASO-13 | 40 | CTGCAAGAAGTAGACTAAAGCATAAAGATAGAGAAAAGGG |
| dZ-2 esASO-14 | 40 | GCTTCAAGGCCAGCAGCAACGAGCAAAAGGTGTGAGTAAA |
| dZ-2 esASO-15 | 40 | CTGTTACAAACAACAACAGCAAGTTGCAAACAAAGTGAAC |
| dZ-2 esASO-16 | 40 | ACCCTTGGAGAGTGCTAGTTGCCATCTCTTTTTGAGGGTT |
| dZ-2 esASO-17 | 40 | ATGATTTTGGAAGCGCTCTGAAAAACAGCAAGAAGTGCAA |
| dZ-2 esASO-18 | 40 | GAAGGAGTAGCATCCTTGATTTCACCTTGCTTCAAAGTTA |
| dZ-2 esASO-19 | 40 | CAGTTCCAATTGTGAAGATTCTCATAAACAAATCCATCCC |
| dZ-3 esASO-1 | 40 | CAAGTACACACTCTGACATTTTAGTAGCAGCAAGATTAGC |
| dZ-3 esASO-2 | 40 | AGAAGCTCTGATTTCTGCAGCTCTAATTAATTGTTGAGTC |
| dZ-3 esASO-3 | 40 | ACATATGTCTGCAAACTTTGAAGTCTGCCTGTGATCAACC |
| dZ-3 esASO-4 | 40 | TATCAATTTGCACTTCAGCCTCAACTTTGTCAAGACGTGA |
| dZ-3 esASO-5 | 40 | AAGGATATCATTTAAAACACTTGAAATTGCACCAAAATTG |
| dZ-3 esASO-6 | 40 | GAGCTAAGTTGTTTAACAAGCGTGTTTAAAGCTTGTGCAT |
| dZ-3 esASO-7 | 40 | TTTGGTTGACCACATCTTGAAGTTTTCCAAGTGCACTTGC |
| dZ-3 esASO-8 | 40 | TGTGGAAGAAAGTGAGTCTTGAATTTTGCCAATAGCACTA |
| dZ-3 esASO-9 | 40 | TTAAATTGGTTGGCAATCAATTTTTGGTTCTCATAGAGAA |
| dZ-3 esASO-10 | 40 | CATTCTGTGTAACTCCAATACCATTAAACCTATAAGCCAT |
| dZ-3 esASO-11 | 40 | TTGCATAGCAAATGGTATTTGTAATGCAGCACCTGCACCA |
| dZ-3 esASO-12 | 40 | AGTAAGGCCGTTAAACTTTTGTGCACAAATGGGGAAACCC |

lsRNA-1 from SARS-CoV-2 *NSP8* 12098/12679. 10-23 DNAzyme: dZ-1.

GGGCCUCAG**AGUUUAGUUCCCUUCCAUCAUAUGCAGCUUUUGCUACUGC**UCA*AGAAGCUU****AU****GAGCAGGCUGUUGCU*AAU**GGUGAUUCUGAAGUUGUUCUUAAAAAGUUGAAGAAGUCUU**UGAAUGUGGCUAAAUCUGAAUUUGACCGUGAUGCAGCCAUGCAACGUAAGUUGGAAAAGAUGGCUGAUCAAGCUAUGACCCAAAUGUAUAAACAGGCUAGAUCUGAGGACAAGAGGGCAAAAGUUACUAGUGCUAUGCAGACAAUGCUUUUCACUAUGCUUAGAAAGUUGGAUAAUGAUGCACUCAACAACAUUAUCAACAAUGCAAGAGAUGGUUGUGUUCCCUUGAACAUAAUACCUCUUACAACAGCAGCCAAACUAAUGGUUGUCAUACCAGACUAUAACACAUAUAAAAAUACGUGUGAUGGUACAACAUUUACUUAUGCAUCAGCAUUGUGGGAAAUCCAACAGGUUGUAGAUGCAGAUAGUAAAAUUGUUCAACUUAGUGAAAUUAGUAUGGACAAUUCACCUAAUUUAGCAUGGCCUCUUAUUGUAACAGCUUUAAGGGCCAAUUCUGCUGUCAAA

lsRNA-2 from SARS-CoV-2 *ORF3a* 25393/26220. 10-23 DNAzyme: dZ-2.

GGGAUGGAUUUGUUUAUGAGAAUCUUCACAAUUGGAACUGUAACUUUGAAGCAAGGUGAAAUCAAGGAUGCUACUCCUUCAG**AUUUUGUUCGCGCUACUGCAACGAUACCGAUACAAGCCUC**ACUC*CCUUUCGG****AU****GGCUUAUUGUUGGCG*UUG**CACUUCUUGCUGUUUUUCAGAGCGCUUCCAAAAUCAUAAC**CCUCAAAAAGAGAUGGCAACUAGCACUCUCCAAGGGUGUUCACUUUGUUUGCAACUUGCUGUUGUUGUUUGUAACAGUUUACUCACACCUUUUGCUCGUUGCUGCUGGCCUUGAAGCCCCUUUUCUCUAUCUUUAUGCUUUAGUCUACUUCUUGCAGAGUAUAAACUUUGUAAGAAUAAUAAUGAGGCUUUGGCUUUGCUGGAAAUGCCGUUCCAAAAACCCAUUACUUUAUGAUGCCAACUAUUUUCUUUGCUGGCAUACUAAUUGUUACGACUAUUGUAUACCUUACAAUAGUGUAACUUCUUCAAUUGUCAUUACUUCAGGUGAUGGCACAACAAGUCCUAUUUCUGAACAUGACUACCAGAUUGGUGGUUAUACUGAAAAAUGGGAAUCUGGAGUAAAAGACUGUGUUGUAUUACACAGUUACUUCACUUCAGACUAUUACCAGCUGUACUCAACUCAAUUGAGUACAGACACUGGUGUUGAACAUGUUACCUUCUUCAUCUACAAUAAAAUUGUUGAUGAGCCUGAAGAACAUGUCCAAAUUCACACAAUCGACGGUUCAUCCGGAGUUGUUAAUCCAGUAAUGGAACCAAUUUAUGAUGAACCGACGACGACUACUAGCGUGCCUUUGUAA

lsRNA-3 from SARS-CoV-2 Spike 24108/24665. 10-23 DNAzyme: dZ-3.

GGGUUUCCCCAUUUGUGCACAAAAGUU**UAACGGCCUUACUGUUUUGCCACCUUUGCUCACAGAUGAA**AUG*AUUGCUCA****AU****ACACUUCUGCACUGU*UAG**CGGGUACAAUCACUUCUGGUUGGACCUUUGGUGCAGGUGC**UGCAUUACAAAUACCAUUUGCUAUGCAAAUGGCUUAUAGGUUUAAUGGUAUUGGAGUUACACAGAAUGUUCUCUAUGAGAACCAAAAAUUGAUUGCCAACCAAUUUAAUAGUGCUAUUGGCAAAAUUCAAGACUCACUUUCUUCCACAGCAAGUGCACUUGGAAAACUUCAAGAUGUGGUCAACCAAAAUGCACAAGCUUUAAACACGCUUGUUAAACAACUUAGCUCCAAUUUUGGUGCAAUUUCAAGUGUUUUAAAUGAUAUCCUUUCACGUCUUGACAAAGUUGAGGCUGAAGUGCAAAUUGAUAGGUUGATCACAGGCAGACUUCAAAGUUUGCAGACAUAUGUGACUCAACAAUUAAUUAGAGCUGCAGAAAUCAGAGCUUCUGCUAAUCUUGCUGCUACUAAAAUGUCAGAGUGUGUACUUG

lsRNA-1 from SARS-CoV-2 *NSP8* 12098/12679. 10-23 DNAzyme: dZ-4.

GGGCCUCAGAGUUUAGUUCCCUUCCAUCAUAUGCAGCUUUUGCUACUGCUCAAGAAGCUUAUGAGCAGGCUGUUGCUAAUGGUGAUUCUGAAGUUGUUCUUAAAAAGUUGAAGAAGUCUUUGAAUGUGGCUAAAUCUGAAUUUGACCGUGAUGCAGCCAUGCAACGUAAGUUGGAAAAGAUGGCUGAUCAAGCUAUGACCCAAAUGUAUAAACAGGCUAGAUCUGAGGACAAGAGGGCAAAAGUUACUAGUGCUAUGCAGACAAUGCUUUUCACUAUGCUUAGAAAGUUGGAUAAUGAUGCACUCAACAACAUUAUCAACAAUGCAAGAGAUGGUUGUGUUCCCUUGAACAUAAUACCUCUUACAACAGCAGCCAAACUAAUGGUUGUCAUACCAGACUAUAACACAUAUAAAAAUACGUGUGAUGGUACAACAUUUACUUAUGCAUCAGCAUUGUGGGAAAUCCAACAGGUUGUAGAUGCAGAUAGUAAAAUUGUUCAACUUAGUGAAAUUAG*UAUGGACA****AU****UCACCUAAUUUAGCA*UGGCCUCUUAUUGUAACAGCUUUAAGGGCCAAUUCUGCUGUCAAA

lsRNA-2 from SARS-CoV-2 *ORF3a* f25393/r26220. 10-23 DNAzyme: dZ-5.

GGGAUGGAUUUGUUUAUGAGAAUCUUCACAAUUGGAACUGUAACUUUGAAGCAAGGUGAAAUCAAGGAUGCUACUCCUUCAGAUUUUGUUCGCGCUACUGCAACGAUACCGAUACAAGCCUCACUCCCUUUCGGAUGGCUUAUUGUUGGCGUUGCACUUCUUGCUGUUUUUCAGAGCGCUUCCAAAAUCAUAACCCUCAAAAAGAGAUGGCAACUAGCACUCUCCAAGGGUGUUCACUUUGUUUGCAACUUGCUGUUGUUGUUUGUAACAGUUUACUCACACCUUUUGCUCGUUGCUGCUGGCCUUGAAGCCCCUUUUCUCUAUCUUUAUGCUUUAGUCUACUUCUUGCAGAGUAUAAACUUUGUAAGAAUAAUAAUGAGGCUUUGGCUUUGCUGGAAAUGCCGUUCC*AAAAACCC****AU****UACUUUAUGAUGCCA*ACUAUUUUCUUUGCUGGCAUACUAAUUGUUACGACUAUUGUAUACCUUACAAUAGUGUAACUUCUUCAAUUGUCAUUACUUCAGGUGAUGGCACAACAAGUCCUAUUUCUGAACAUGACUACCAGAUUGGUGGUUAUACUGAAAAAUGGGAAUCUGGAGUAAAAGACUGUGUUGUAUUACACAGUUACUUCACUUCAGACUAUUACCAGCUGUACUCAACUCAAUUGAGUACAGACACUGGUGUUGAACAUGUUACCUUCUUCAUCUACAAUAAAAUUGUUGAUGAGCCUGAAGAACAUGUCCAAAUUCACACAAUCGACGGUUCAUCCGGAGUUGUUAAUCCAGUAAUGGAACCAAUUUAUGAUGAACCGACGACGACUACUAGCGUGCCUUUGUAA

Figure S1. Sequences of *in-vitro* transcribed SARS-CoV-2 lsRNA with corresponding 10-23 DNAzyme binding site (underlined) and cleavage site (bolded and underlined). The hybridization region for the rationally designed antisense DNA oligonucleotides (RD ASO) colored red for the upstream RD ASO and blue for the downstream RD ASO. All sequences are in the 5’ to 3’ direction.

**
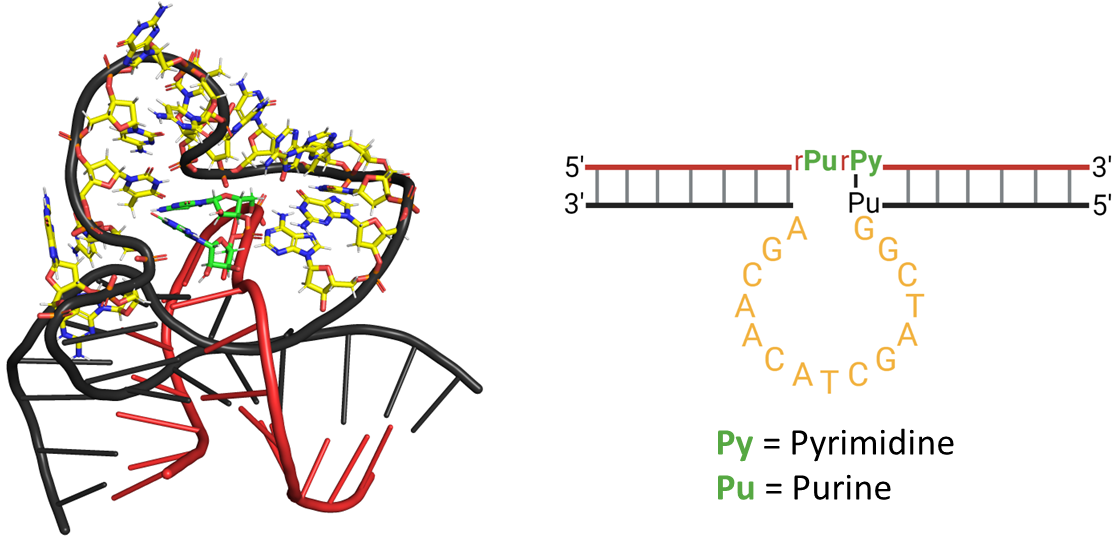

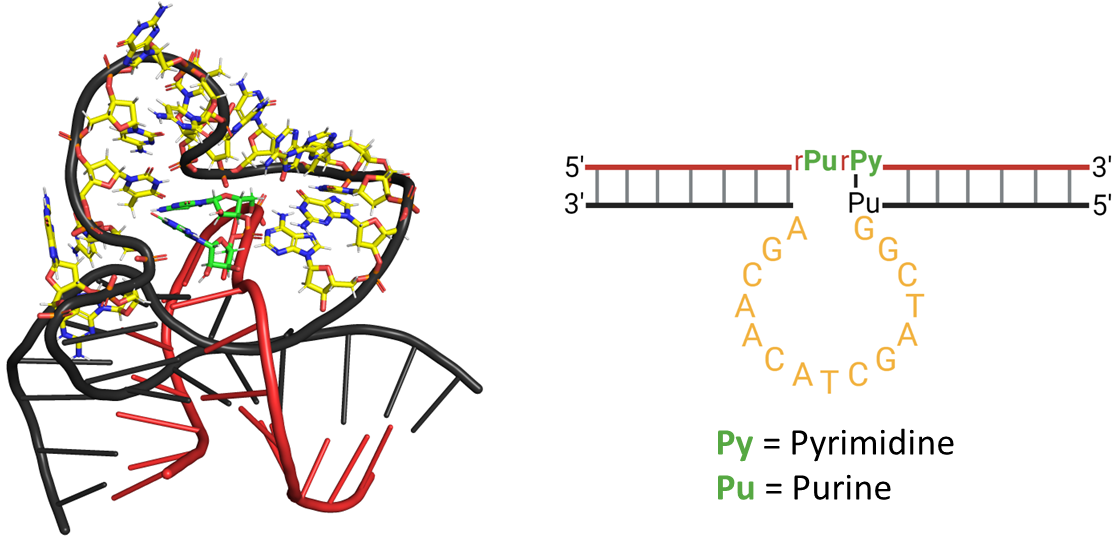
­­­­**

Figure S2: Tertiary (**L**) and secondary (**R**) structures of the 10-23 DNAzyme**.** It has a conserved catalytic loop (yellow) that cleaves RNA (red) at bound pyrimidine (Py) and unbound purine (Pu) linkage (green). DNA binding arms (black) can be designed to hybridize to any RNA molecule of interest. The 10-23 DNAzymes used in this study possessed 5′ and 3′ binding arm lengths of 15-nt and 8-nt respectively. The tertiary structure was elucidated using nuclear magnetic resonance imaging by Borggräfe and colleagues (PDB ID: 7PDU)^1^.

**
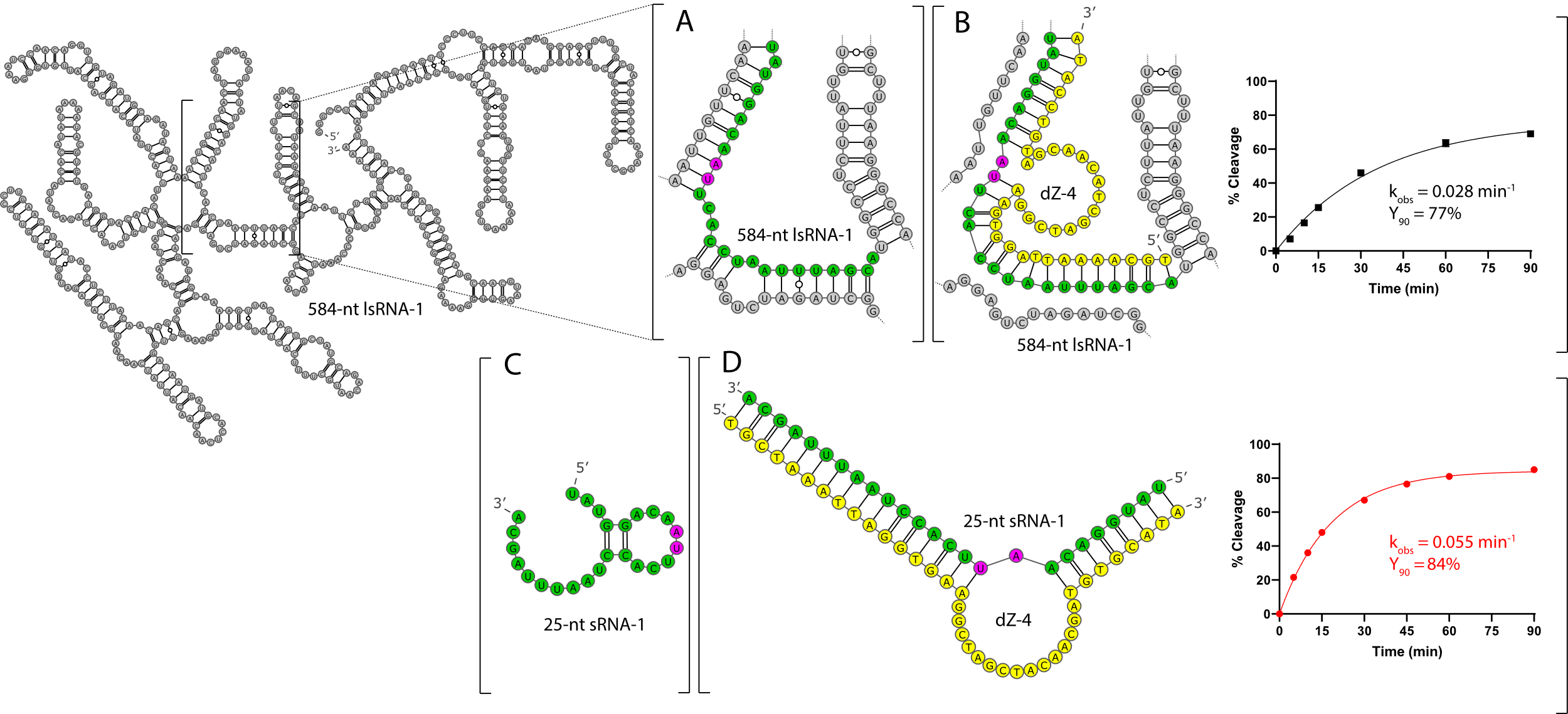
**

**­­**Figure S3. RNA cleavage activity comparison of a 10-23 DNAzyme, dZ-4, with lsRNA-1 (A & B) and short sRNA-4 substrate (C & D). The dZ-4 target site is shown in green with the AU cleavage site shown in pink and dZ-4 shown in yellow.­­ Experimental conditions are described in the Materials and Methods section.

**
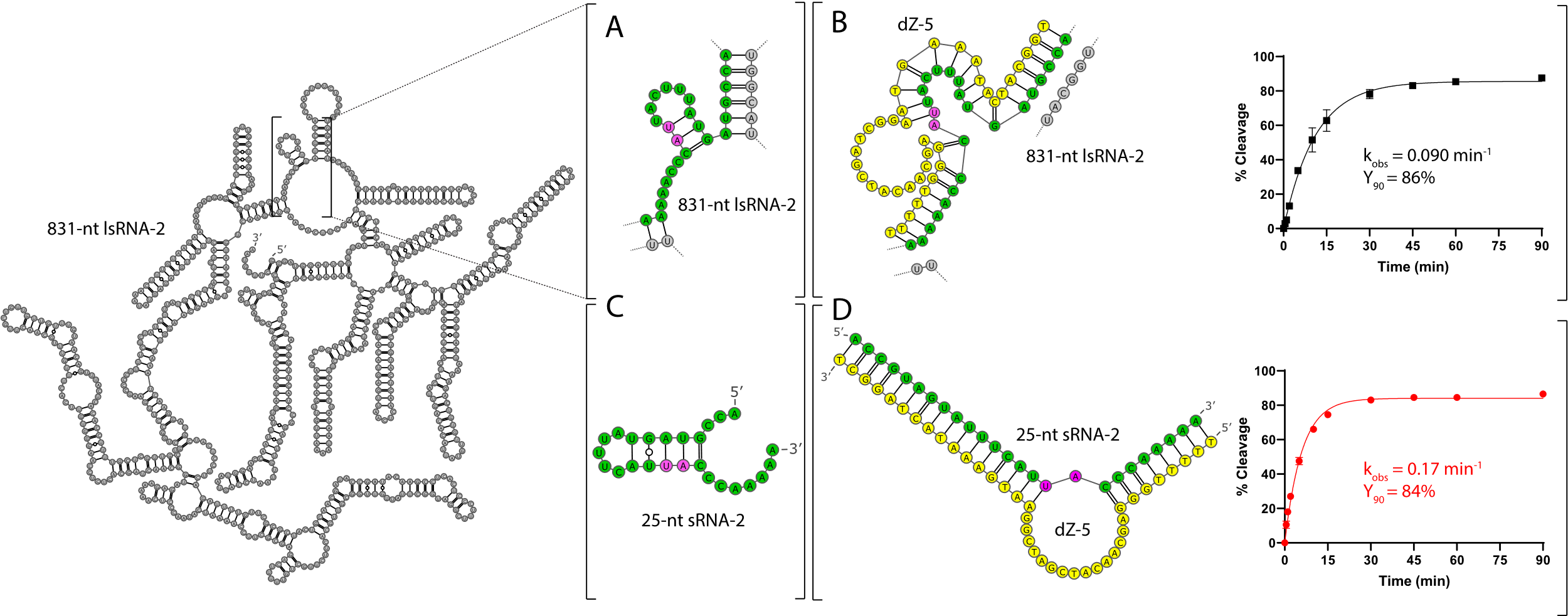
**

Figure S4. RNA cleavage activity comparison of a 10-23 DNAzyme, dZ-5, with lsRNA-2 (A & B) and short sRNA-5 substrate (C & D). The dZ-5 target site is shown in green with the AU cleavage site shown in pink and dZ-5 shown in yellow. Experimental conditions are described in the Materials and Methods section.


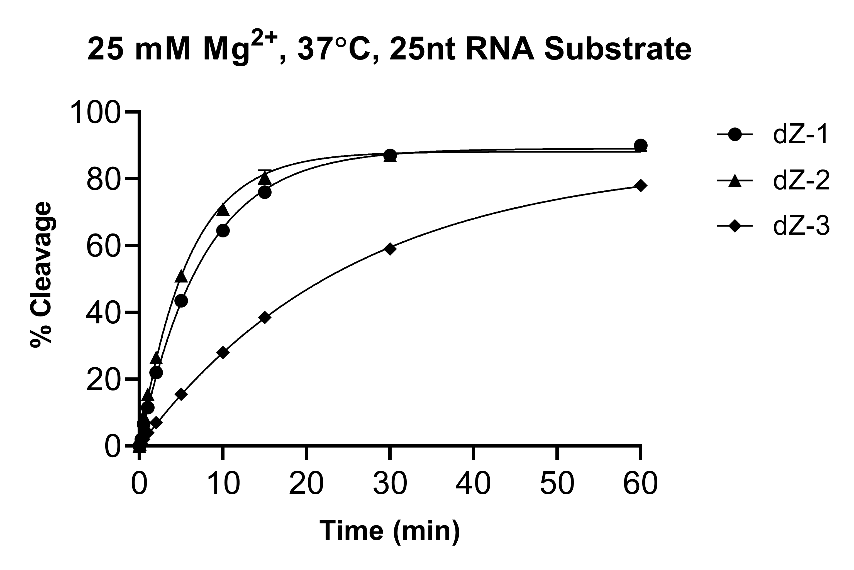


*k*_obs_ = 0.17 min^-1^

*k*_obs_ = 0.39 min^-1^

*k*_obs_ = 0.13 min^-1^

Figure S5. Kinetic analysis of dZ-1 (circles), dZ-2 (triangles) and dZ-3 (diamonds) with their respective short, 25-nt RNA (sRNA) substrates under optimized conditions. Experimental conditions are described in the Materials and Methods section.

­­­


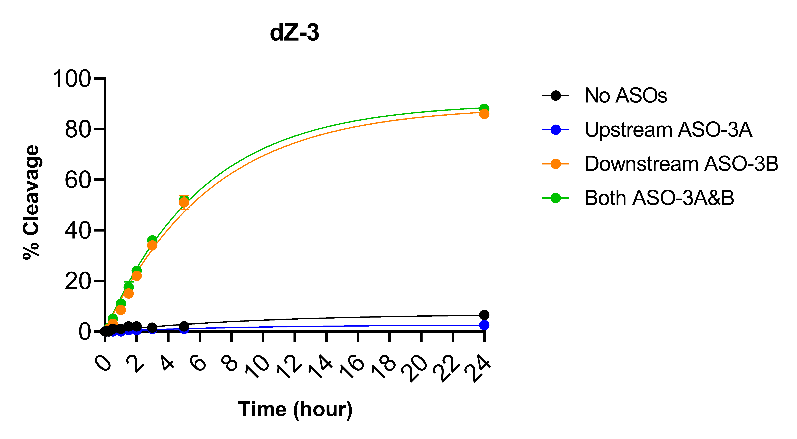

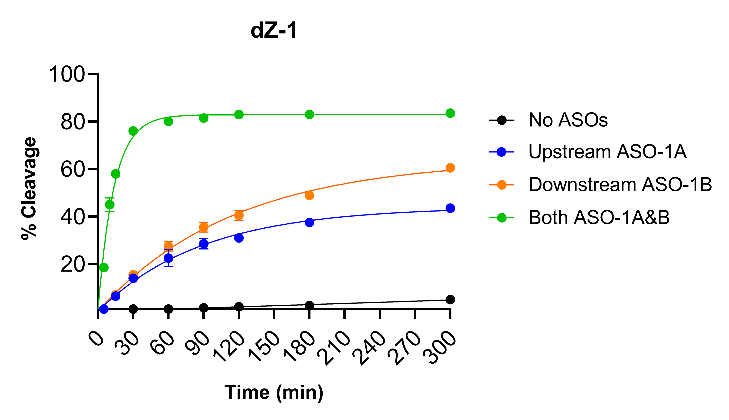

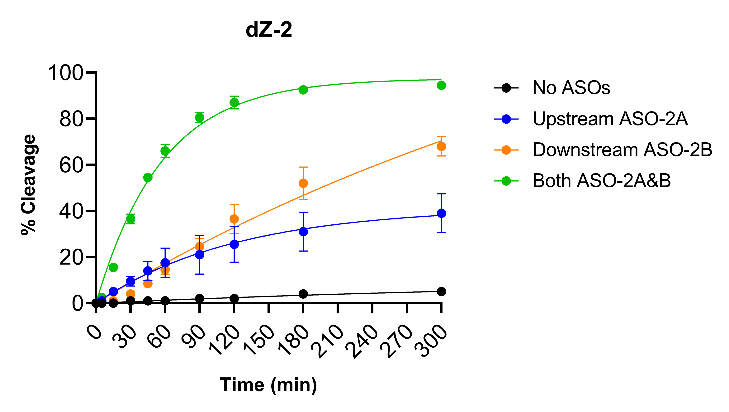


**C**

**B**

**A**

|  | Y_90_ no ASOs (%) | Y_90_ Upstream ASO A (%) | Y_90_ Downstream ASO B (%) | Y_90_ ASO A&B (%) |
| --- | --- | --- | --- | --- |
| dZ-1 | **2** | **29** | **36** | **82** |
| dZ-2 | **2** | **21** | **25** | **81** |
| dZ-3 | **2** | **1** | **15** | **18** |

|  | *k*_obs_ no ASOs (min^-1^) | *k*_obs_ Upstream ASO A (min^-1^) | *k*_obs_ Downstream ASO B (min^-1^) | *k*_obs_ ASO A&B (min^-1^) |
| --- | --- | --- | --- | --- |
| dZ-1 | **0.000039** | **0.011** | **0.0086** | **0.078** |
| dZ-2 | **0.00016** | **0.0082** | **0.0018** | **0.017** |
| dZ-3 | **0.000063** | **0.000021*** | **0.0025** | **0.0027** |

Figure S6. Kinetic time-course reactions with dZ-1, dZ-2, and dZ-3 10-23 DNAzymes with their respective lsRNA substrates and rationally designed ASOs added separately. Comparison in 10-23 DNAzyme activity was evaluated using Y_90_ and *k*_obs_ values for each reaction. For dZ-1 (A) and dZ-2 (B), both rationally designed ASOs are required for optimal activity, but for dZ-3 (C), only the downstream ASO-3B is required. Experimental conditions are described in the materials and methods section. * Indicates *k*_obs_ obtained from a linear fit.

lsRNA-1 from SARS-CoV-2 *NSP8* 12098/12679. 10-23 DNAzyme: dZ-1.

**GGGCCUCAGAGUUUAGUUCCCUUCCAUCAUAUGCAGCUUU**_UGCUACUGCUCA*AGAAGCUU****AU****GAGCAGGCUGUUGCU*AAUGGUGAUUCUGAAGUUGUUCUUAAA_**AAGUUGAAGAAGUCUUUGAAUGUGGCUAAAUCUGAAUUUG**_**ACCGUGAUGCAGCCAUGCAACGUAAGUUGGAAAAGAUGGC**_**UGAUCAAGCUAUGACCCAAAUGUAUAAACAGGCUAGAUCU**_**GAGGACAAGAGGGCAAAAGUUACUAGUGCUAUGCAGACAA**_**UGCUUUUCACUAUGCUUAGAAAGUUGGAUAAUGAUGCACU**_**CAACAACAUUAUCAACAAUGCAAGAGAUGGUUGUGUUCCC**_**UUGAACAUAAUACCUCUUACAACAGCAGCCAAACUAAUGG**_**UUGUCAUACCAGACUAUAACACAUAUAAAAAUACGUGUGA_UGGUACAACAUUUACUUAUGCAUCAGCAUUGUGGGAAAUC_CAACAGGUUGUAGAUGCAGAUAGUAAAAUUGUUCAACUUA_GUGAAAUUAGUAUGGACAAUUCACCUAAUUUAGCAUGGCC_UCUUAUUGUAACAGCUUUAAGGGCCAAUUCUGCUGUCAAA**

lsRNA-2 from SARS-CoV-2 *ORF3a* 25393/26220. 10-23 DNAzyme: dZ-2.

**GGGAUGGAUUUGUUUAUGAGAAUCUUCACAAUUGGAACUG**_**UAACUUUGAAGCAAGGUGAAAUCAAGGAUGCUACUCCUUC**_AGAUUUUGUUCGCGCUACUGCAACGAUACCGAUACAAGCCUCACUC*CCUUUCGG****AU****GGCUUAUUGUUGGCG***UUGCACUUCUUGCUGUUUUUCAGAGCGCUUCCAAAAUCAU**_**AACCCUCAAAAAGAGAUGGCAACUAGCACUCUCCAAGGGU**_**GUUCACUUUGUUUGCAACUUGCUGUUGUUGUUUGUAACAG**_**UUUACUCACACCUUUUGCUCGUUGCUGCUGGCCUUGAAGC**_**CCCUUUUCUCUAUCUUUAUGCUUUAGUCUACUUCUUGCAG**_**AGUAUAAACUUUGUAAGAAUAAUAAUGAGGCUUUGGCUUU**_**GCUGGAAAUGCCGUUCCAAAAACCCAUUACUUUAUGAUGC**_**CAACUAUUUUCUUUGCUGGCAUACUAAUUGUUACGACUAU**_**UGUAUACCUUACAAUAGUGUAACUUCUUCAAUUGUCAUUA**_**CUUCAGGUGAUGGCACAACAAGUCCUAUUUCUGAACAUGA**_**CUACCAGAUUGGUGGUUAUACUGAAAAAUGGGAAUCUGGA**_**GUAAAAGACUGUGUUGUAUUACACAGUUACUUCACUUCAG**_**ACUAUUACCAGCUGUACUCAACUCAAUUGAGUACAGACAC**_**UGGUGUUGAACAUGUUACCUUCUUCAUCUACAAUAAAAUU**_**GUUGAUGAGCCUGAAGAACAUGUCCAAAUUCACACAAUCG**_**ACGGUUCAUCCGGAGUUGUUAAUCCAGUAAUGGAACCAAU**_**UUAUGAUGAACCGACGACGACUACUAGCGUGCCUUUGUAA**

lsRNA-3 from SARS-CoV-2 Spike 24108/24665. 10-23 DNAzyme: dZ-3.

**GGGUUUCCCCAUUUGUGCACAAAAGUUUAACGGCCUUACU**_GUUUUGCCACCUUUGCUCACAGAUGAAAUG*AUUGCUCA****AU****ACACUUCUGCACUGU*UAGCGGGUACAAUCACUUCUGGUUGGACCUU_**UGGUGCAGGUGCUGCAUUACAAAUACCAUUUGCUAUGCAA**_**AUGGCUUAUAGGUUUAAUGGUAUUGGAGUUACACAGAAUG**_**UUCUCUAUGAGAACCAAAAAUUGAUUGCCAACCAAUUUAA**_**UAGUGCUAUUGGCAAAAUUCAAGACUCACUUUCUUCCACA**_**GCAAGUGCACUUGGAAAACUUCAAGAUGUGGUCAACCAAA**_**AUGCACAAGCUUUAAACACGCUUGUUAAACAACUUAGCUC**_**CAAUUUUGGUGCAAUUUCAAGUGUUUUAAAUGAUAUCCUU**_**UCACGUCUUGACAAAGUUGAGGCUGAAGUGCAAAUUGAUA**_**GGUUGATCACAGGCAGACUUCAAAGUUUGCAGACAUAUGU**_**GACUCAACAAUUAAUUAGAGCUGCAGAAAUCAGAGCUUCU**_**GCUAAUCUUGCUGCUACUAAAAUGUCAGAGUGUGUACUUG**

Figure S7. Sequences of *in-vitro* transcribed SARS-CoV-2 RNA with corresponding 10-23 DNAzyme binding site (underlined) and cleavage site (bolded and underlined). The hybridization region for the experimental screening antisense DNA oligonucleotides (esASOs) are bolded and separated by the underscore (_). The sequences of esASOs for each 10-23 DNAzyme are shown in Table S1. All sequences are in the 5’ to 3’ direction.


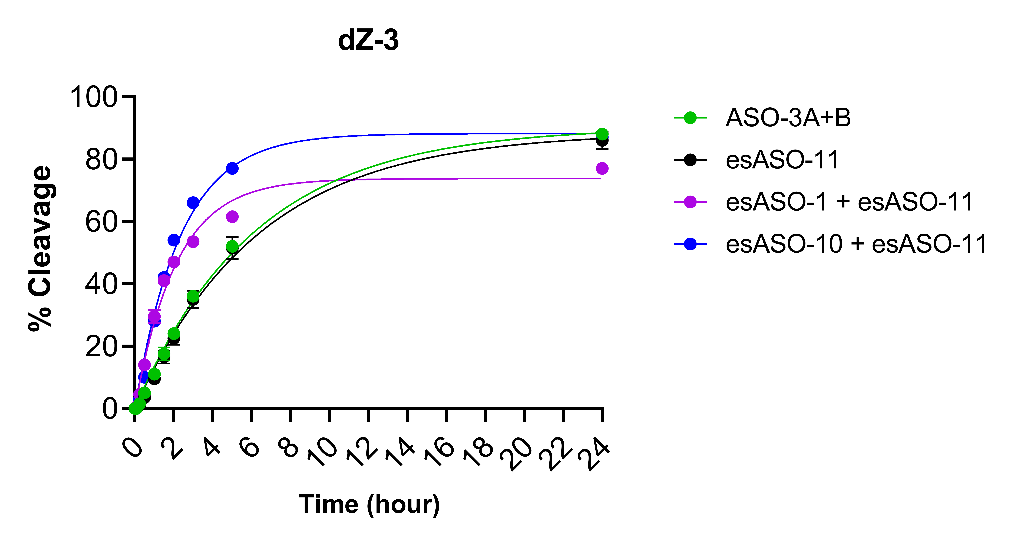


|  | ASO-3A+B | esASO-11 | esASO-1 + esASO-11 | esASO-10 + esASO-11 |
| --- | --- | --- | --- | --- |
| Y_90_ (%) | **18** | **17** | **41** | **42** |
| *k*_obs_ (min^-1^) | **0.0027** | **0.0027** | **0.0078** | **0.0070** |

Figure S8. Kinetic time-course reactions testing various combinations of experimental screening ASOs (esASOs) on dZ-3 accessibility for lsRNA-3. Comparison in dZ-3 activity was evaluated using *k*_obs_ and Y_90_ values for each reaction. Both combinations of esASOs (esASO-1 + esASO-11 and esASO-10 + esASO-11) offered significant improvement in dZ-3 accessibility for lsRNA-3 compared to the rationally designed ASO-3A+B and esASO-11 reactions. Experimental conditions are described in the materials and methods section.


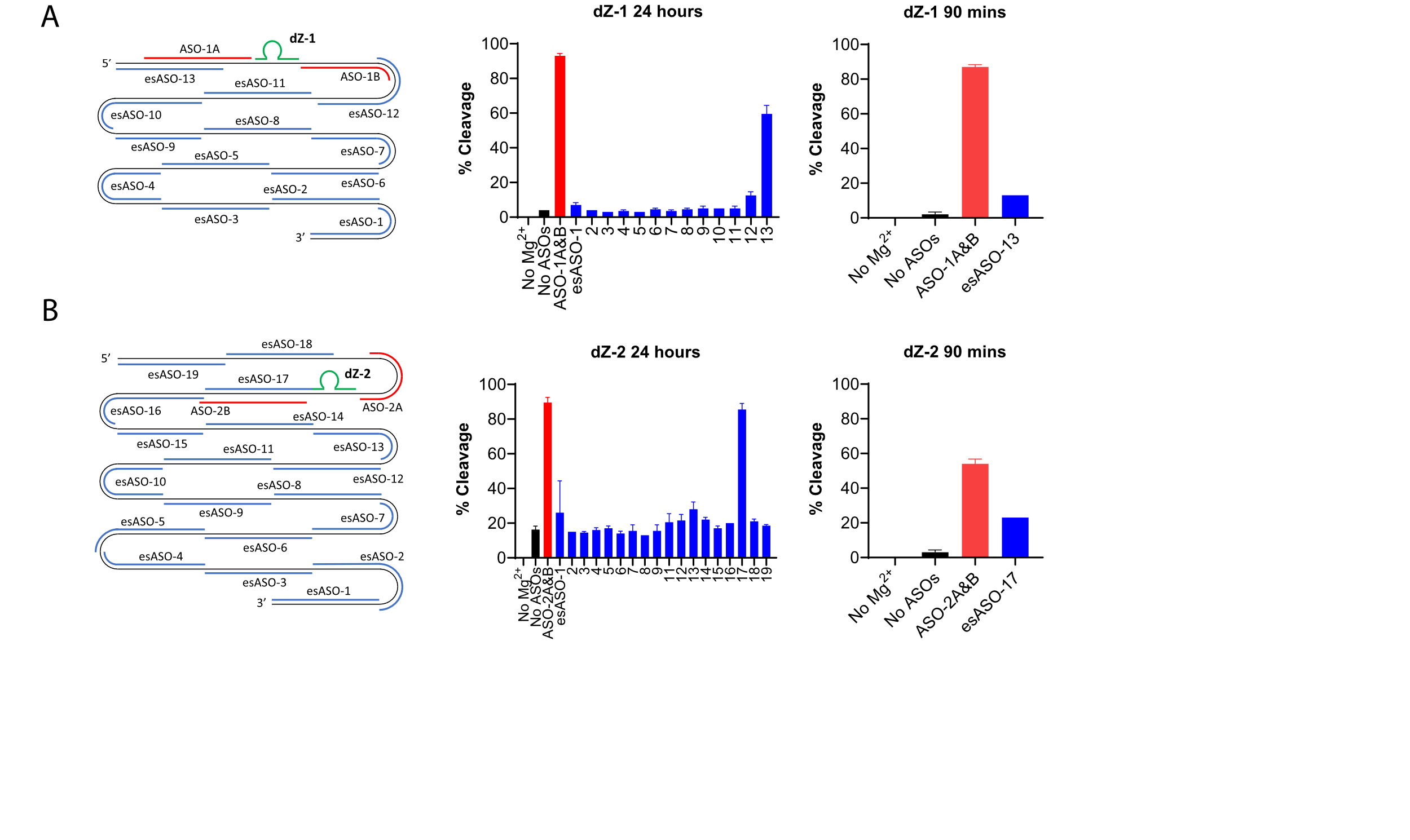


Figure S9. Experimental ASO screen for dZ-1 (A) and dZ-2 (B) with experimental screening ASO (esASO) binding sites within lsRNA-1 and lsRNA-2 respectively. Each screening reaction contained equimolar amounts of DNAzyme (green) and 40-nt esASO (blue) starting with esASO-1 from the 3’-end of the respective lsRNA substrate, with 13 esASOs total needed to cover the entire sequence space of lsRNA-1, and 19 esASOs total for lsRNA-2. After 24 hours, esASO-13 was found to elicit the most significant enhancment in dZ-1 activity for lsRNA-1 and similarly, esASO-17 for dZ-2 activity against lsRNA-2. For both dZ-1 and dZ-2, the rationally designed ASOs provided a greater enhancement in DNAzyme activity than any esASO. Experimental conditions are described in materials and methods.

**
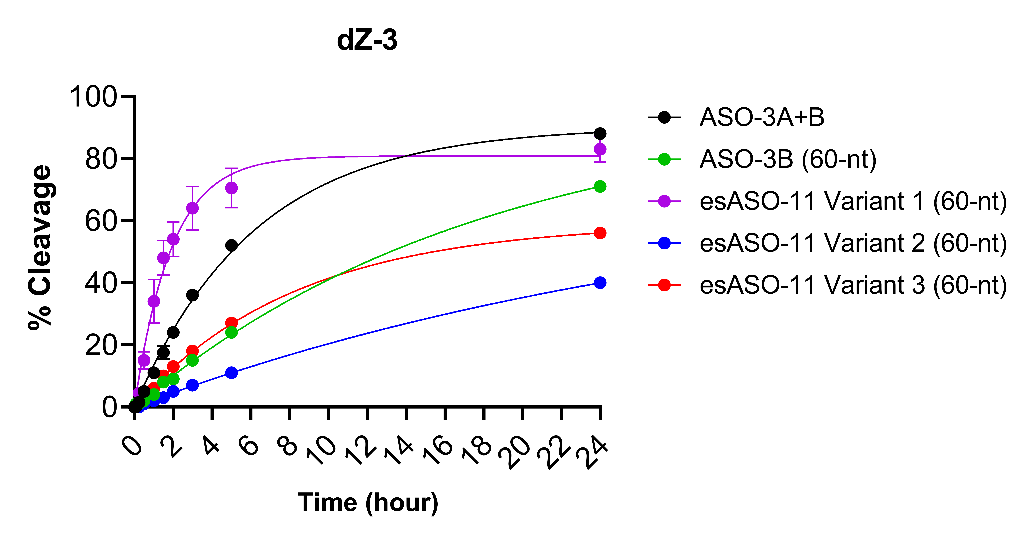
**

|  | ASO-3A+B | ASO-3B (60-nt) | esASO-11 Variant 1 (ASO-V1) (60-nt) | esASO-11 Variant 2 (60-nt) | esASO-11 Variant 3 (60-nt) |
| --- | --- | --- | --- | --- | --- |
| Y_90_ (%) | **18** | **8** | **48** | **3** | **10** |
| *k*_obs_ (min^-1^) | **0.0027** | **0.00092** | **0.0087** | **0.00052** | **0.0020** |

Figure S10. Kinetic time-course reactions testing various combinations of extended experimental screening ASOs (esASOs) on dZ-3 accessibility for lsRNA-3. Comparison in dZ-3 activity was evaluated using *k*_obs_ and Y_90_ values for each reaction. A total of 4 different extended esASOs were tested with dZ-3 for lsRNA-3 cleavage, each 60-nt in length, including a 20-nt extension to the 3’ end of ASO-3B, which showed limited effectiveness. The remaining 3 extended esASOs were variants of esASO-11 and included variant 1, which was extended 20-nt on the 3’ end of esASO-11, variant 2, which was extended 20-nt on the 5’ end of esASO-11, and variant 3, which possessed an additional 10-nt to both the 5’ and 3’ ends of esASO-11. Variant 1 (ASO-V1) showed the greatest improvement in dZ-3 activity for lsRNA-3. Experimental conditions are described in materials and methods.

**References**

1. Borggräfe, J. *et al.* Time-resolved structural analysis of an RNA-cleaving DNA catalyst. *Nature* (2021) doi:10.1038/s41586-021-04225-4.
